# Multi-omics reveals mechanism of Qi-Po-Sheng-Mai granule in reducing atrial fibrillation susceptibility in aged rats

**DOI:** 10.1186/s13020-025-01154-6

**Published:** 2025-09-03

**Authors:** Shuqing Shi, Xiaohan Zhang, Jiayu Lv, Zhenyue Fu, Yajiao Wang, Yihang Du, Chenglin Duan, Huan Wang, Bai Du, Qingqiao Song, Yuanhui Hu

**Affiliations:** 1 Department of Internal Medicine, Guang’anmen Hospital, China Academy of Chinese Medical Sciences, Beijing, China; 2 China Academy of Chinese Medical Sciences, Beijing, China; 3 Research Center of Clinical Epidemiology, Peking University Third Hospital, Beijing, PR China; 4 Beijing University of Chinese Medicine, Beijing, China; 5 Department of Cardiovascular, Guang’anmen Hospital, China Academy of Chinese Medical Sciences, 5 Beixiange Street, Xicheng District, Beijing, 100053 People’s Republic of China

**Keywords:** Atrial fibrillation, Metabolomics, Transcriptomics, Qi-Po-Sheng-Mai granule, Nampt/NAD^+^

## Abstract

**Background:**

Atrial Fibrillation (AF) is the most common arrhythmia in clinical practice, and age is an independent risk factor for the development of AF. Qi-Po-Sheng-Mai granule (QPSM) has been used clinically to treat aging-related AF, however, its underlying mechanisms remain incompletely understood.

**Methods:**

In this study, we established a D-galactose-induced aging rat model to evaluate the effects of QPSM on aging-related AF through electrocardiograms, echocardiography, and histopathological analysis. Further, we employed transcriptomics and metabolomics to uncover molecular mechanisms and targets. Finally, in vivo experiments were conducted to validate the expression of key targets in the D-Gal-induced aging rat model and the intervention effects of QPSM.

**Results:**

QPSM significantly reduced the susceptibility to AF in aging rats and alleviated atrial dilation and fibrosis. The combined analysis of transcriptomics and metabolomics suggested that QPSM may inhibit the occurrence of aging-related AF by modulating Nampt expression and increasing NAD^+^ content in atrial tissue. Additionally, in vivo experiments confirmed that QPSM increased ATP content, reduced mitoSOX fluorescence intensity, and decreased the proportion of senescent cells. Whole-cell patch clamp results showed that QPSM could prolong the action potential duration of isolated atrial cells, increase *I*_caL_. This might be achieved by regulating the expression of Oxi-CaMKII and RyR_2_^ser2814^, thereby alleviating calcium overload in atrial cells.

**Conclusions:**

Our study demonstrates that QPSM may reduce the susceptibility to aging-related AF by regulating Nampt expression and NAD^+^ content, thereby mitigating calcium overload in atrial cells. This provides a direction for future research in related fields.

**Graphical Abstract:**

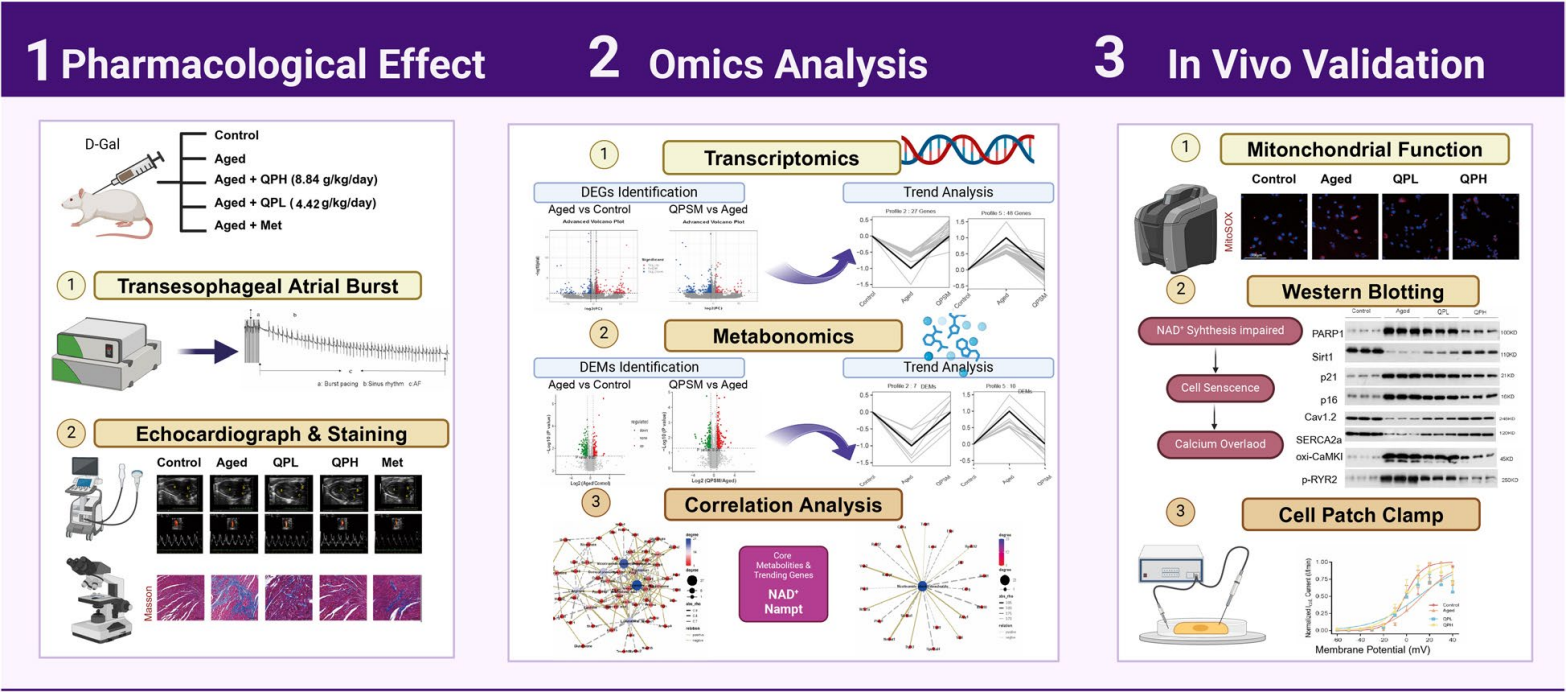

**Supplementary Information:**

The online version contains supplementary material available at 10.1186/s13020-025-01154-6.

## Introduction

Atrial fibrillation (AF) is a common cardiac arrhythmia characterized by disrupted electrical impulses in the atrium, resulting in irregular contractions. Age is an independent predictor of the onset and progression of AF [1–3]. Epidemiological studies indicate that the prevalence of AF increases markedly with age, rising from 0.5% in individuals aged 50–59 to nearly 9% in those aged 80–89 [4]. AF significantly elevates the risk of ischemic stroke [5], heart failure [6, 7], and myocardial infarction [6], imposing a substantial economic burden on the healthcare system. In recent years, the development of novel antiarrhythmic drugs like vernakalant and nifekalant, along with advancements in radiofrequency ablation technology, has provided more treatment options for patients with AF [8, 9]. However, these treatments are accompanied by risks of arrhythmogenicity, severe extracardiac side effects, and high AF recurrence rates. Consequently, current treatment modalities for AF are hindered by inadequate efficacy and safety.

In recent years, risk factor management has gained prominence in addressing the growing burden of AF and is now positioned by the European Society of Cardiology guidelines as the third pillar of the integrated ABC pathway, after anticoagulation and rate/rhythm control therapy [8]. Risk factor management is usually categorized into modifiable (hypertension, diabetes, smoking, obesity, etc.) and non-modifiable risk factors (age, race, gender, etc.). For example, significant weight loss was followed by a significant reduction in AF load, improving outcomes. In contrast, increasing age, masculinity, and genetic predisposition have traditionally been considered the major non-modifiable risk factors for AF [10]. Recent research suggests that while physiological aging is an irreversible risk factor for AF, slowing biological aging can reduce the incidence of aging-related AF [10]. Mehdizadeh et al. provide evidence indicating that the use of the senolytic agents quercetin and dasatinib to prevent cellular senescence eliminates the inducibility of AF in rats post-myocardial infarction [11]. Metformin, an AMP-activated protein kinase (AMPK) activator, is a notable anti-aging drug that has been proven in numerous studies to reduce AF susceptibility independently of its hypoglycemic effect [12–14]. Therapies that effectively mitigate or reverse biological aging may emerge as potent preventive strategies for AF, with the promotion of healthy aging recognized as a fundamental preventive objective [15, 16]. Mounting evidence indicates that cellular senescence contributes to the pathophysiology of AF, encompassing changes such as myocardial hypertrophy, altered electrical activity, calcium overload, and metabolic remodeling [10].

Notably, traditional chinese medicine (TCM), with its multi-target integrative effects from diverse active compounds, has been widely used in China to treat aging-related AF, particularly for maintaining sinus rhythm and reducing new ischemic stroke incidence, providing a promising foundation for drug development in this field [17–19]. The classic treatise of TCM Huang Di Nei Jing (The Yellow Emperor's Classic of Internal Medicine), asserts that "after 40 years of age, yin qi is reduced by half", and outlines the pathology of aging as marked by deficiencies in qi and yin. This deficiency forms basis for the onset of AF in the elderly. Qi-Po-Sheng-Mai Granule (QPSM), originating from the traditional formula ShengMai San, primarily consits of *Astragali Radix* (HuangQi), *Succinum* (HuPo), *Ophiopogonis Radix* (MaiDong), *Schisandrae Chinensis Fructus* (WuWeiZi), *Glehniae Radix* (BeiShaShen) and *Ziziphi Spinosae Semen* (SuanZaoRen) et al. which is beneficial for enhancing both qi and yin [20]. A clinical study involving elderly AF patients over 70 demonstrated that QPSM was as effective as amiodarone in decreasing the frequency of AF episodes and extending sinus rhythm after 12-week treatment. In our prior mechanistic investigation, QPSM exhibited potent antiarrhythmic properties in an acetylcholine-calcium chloride (Ach-CaCl₂)-induced AF model. Critical analysis revealed that QPSM attenuates pathological atrial remodeling through dual modulation of calcium regulatory machinery: it enhances the expression of L-type calcium channel subunit *CACNA1C* and sarcoplasmic reticulum Ca^2^⁺-ATPase *SERCA2a* while concurrently inhibiting calcium/calmodulin-dependent kinase II (*CAMKII*) and sodium-calcium exchanger *NCX1* activity. This calcium homeostatic regulation was corroborated by integrated multi-omics approaches, including network pharmacology and single-nucleus transcriptomic profiling, which identified calcium signaling as the dominant pathway mediating QPSM's therapeutic effects [20]. Expanding upon these mechanistic insights, the present study interrogates QPSM's capacity to mitigate age-associated AF pathogenesis, with particular emphasis on nicotinamide adenine dinucleotide (NAD⁺) metabolic reprogramming and senescence-associated phenotypic alterations.

With the advancement of high-throughput sequencing technology, systems biology-driven omics approaches (genomics, transcriptomics, proteomics, metabolomics, lipidomics, and other omics methods) fulfill the requirements of TCM research, enabling the decoding of complex components, targets, and drug-disease interactions [21, 22]. In this study, we utilized transcriptomics and metabolomics, along with in vivo experiments, to explore the molecular mechanisms and potential targets of QPSM for aging-related AF.

## Materials and methods

### Materials

QPSM was supplied by the Guang'anmen Hospital Preparation Center, Chinese Academy of Traditional Chinese Medicine, with its detailed preparation process described in a prior study [20]. Ultra Performance Liquid Chromatography (Thermo Vanquish UHPLC, Thermo Fisher Scientific, MA, USA) coupled with a high-resolution mass spectrum (Q-Exactive HF, Thermo Fisher Scientific, MA, USA) were used to analyze the QPSM chemical identification. Detailed reagents and some of the methods were outlined in the Supplementary File 1.

### Experimental animals

**Fig. 1:**
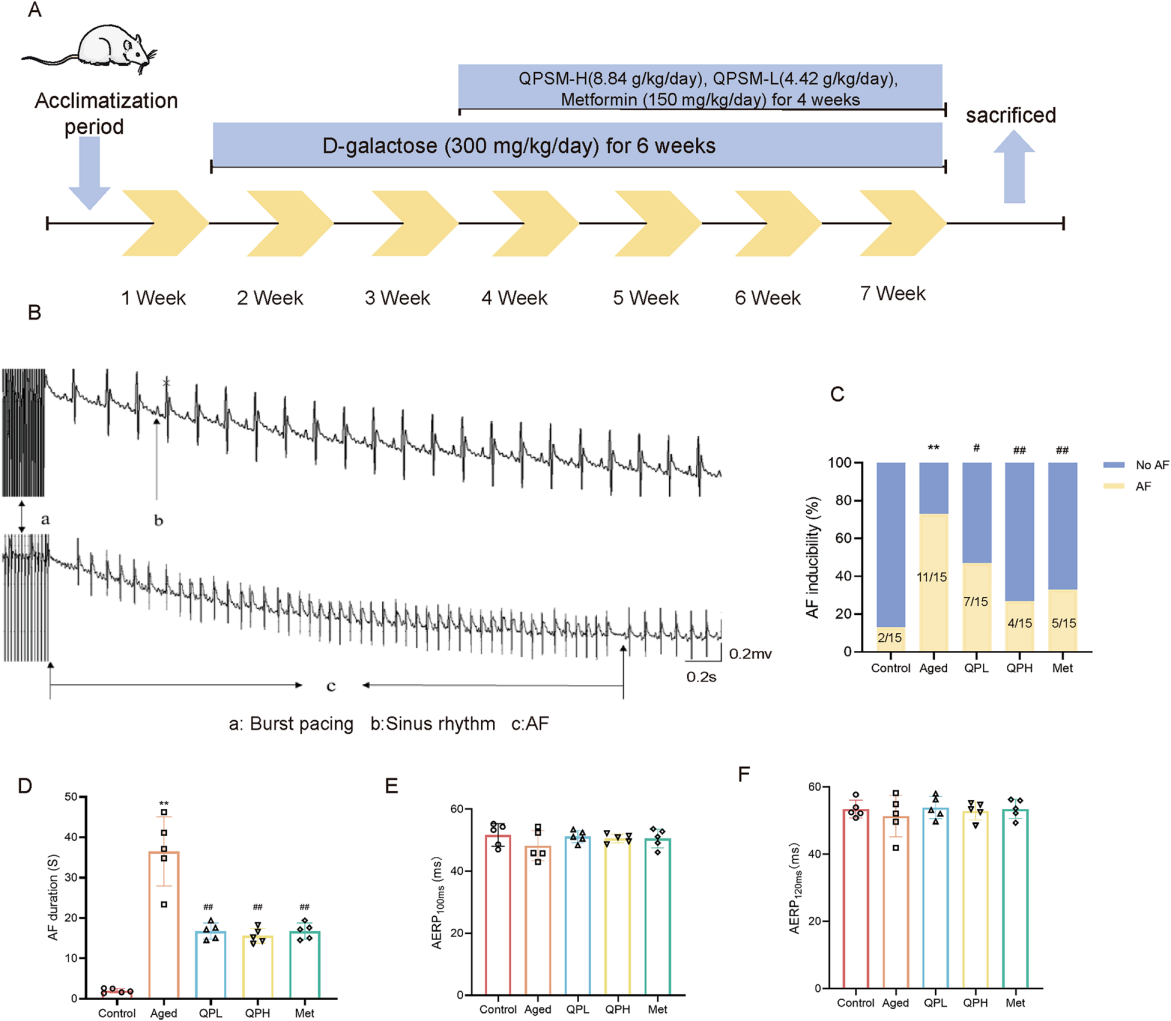
QPSM Reduced the Susceptibility to AF in Aging Rat. **A** Experimental procedure schematic for QPSM treatment of aging-related AF. **B** The representative surface electrocardiogram after the transesophageal atrial burst pacing. **C** The AF inducibility in the five study groups (Fisher test, *n* = 15). **D** AF duration (Mann–Whitney rank-sum test, *n* = 15). **E** AERP_100ms_. **F** AERP_120ms_. ^*^p < 0.05 and ^**^p < 0.01 compared with the Control group, ^#^*p* < 0.05 and ^##^*p* < 0.01 compared with the Aged group

Sprague Dawley (SD) rats (Male, 8 weeks) were obtained from Beijing WeiTong LiHua Laboratory Animal Technology Co. and housed in the SPFII Laboratory Animal Center of Guang'anmen Hospital.Based on previous studies [23, 24], we developed an aging rat model by subcutaneously injecting 300 mg/kg/day D-galactose at the back of the neck for 6 weeks. A control group was administered an equivalent volume of saline through subcutaneous injection. Following a 1-week acclimatisation and feeding phase, the rats were randomly assigned to 5 groups: control group, aged group, high-QPSM (QPH, 8.84 g/kg/day) treatment group for aging, low-QPSM (QPL, 4.42 g/kg/day) treatment group for aging, and metformin (Met, 150 mg/kg/day) treatment group for aging. Gavage commenced 1 week following the initial subcutaneous injection of D-galactose, with dosages of QPSM and Met determined from previous studies [10, 20, 25]. The control group and the aging group were administered saline via gavage. All groups received gavage delivery for five successive weeks, as seen in Fig. 1A. Subsequently, we performed electrophysiological investigation and echocardiography, as detailed in Supplementary File 2.

### Transcriptomics and bioinformatic analysis and Reverse Transcription Quantitative Polymerase Chain Reaction (RT-qPCR) analysis

Three rats in each control, aging, and QPH group were anaesthetised intraperitoneally with 2% sodium pentobarbital (0.33 mL/100 g). Trizol was utilised to isolate and purify RNA from the entire sample. The Bioanalyzer 2100 and RNA 6000 Nano LabChip Kit were employed to evaluate the total RNA quantity. Thereafter, oligo(dT) magnetic beads were employed for two purification rounds to selectively isolate polyA-containing mRNA. The isolated mRNA was subsequently fragmented, and the resultant RNA fragments were reverse transcribed into cDNA. The isolated double-stranded DNA fragments were subjected to end repair, A-base addition, and ligation of Illumina sequencing adapters. Lastly, the Illumina Novaseq 6000 was used to perform paired-end sequencing with a mode of PE150 in accordance with standard procedures. Transcriptomic analysis was provided by LC-Bio Technology CO., Ltd. (Hangzhou, China).

DESeq2 software was employed to analyze the differentially expressed genes (DEGs) between two distinct groups. Genes with a false discovery rate (FDR) below 0.05 and an absolute fold change (FC) ≥ 1.5 or ≤ 1/1.5 were identified as DEGs [26]. These DEGs were subsequently subjected to enrichment analysis for Gene Ontology (GO) functions and Kyoto Encyclopedia of Genes and Genomes (KEGG) pathways. Gene Set Enrichment Analysis (GSEA v4.1.0) and the Molecular Signatures Database (MSigDB) were utilized to determine if the two groups exhibited differences in specific GO terms and KEGG pathways [27]. A gene expression matrix was input, and genes were ranked using the Signal2Noise normalization method. GO terms and KEGG pathways with |NES|> 1, NOM p < 0.05, and FDR q < 0.25 were considered significantly distinct between the two groups. Total RNA was extracted from cardiac tissue and reverse transcribed into cDNA for RT-qPCR analysis to verify the transcriptome results. Total RNA was isolated using TRIzol (Thermo Scientific, USA) following the manufacturer's protocol. For cDNA synthesis, 1 μg RNA was reverse-transcribed with Revert Aid cDNA Synthesis Kit (Thermo Scientific, USA). SYBR Green Master Mix (KAPA Biosystems, USA) and Step One System (Applied Biosystems, USA) were employed for triplicate RT-qPCR amplifications. Gene expression levels normalized to β-actin were calculated using the 2^(-ΔΔCt) method. Primer sequences are listed in Table 1.

**Table 1:** Primer sequences used for RT-qPCR

Gene symbol	Forward primer (5' → 3')	Reverse primer (5' → 3')
*Gpm6a*	CCTGGGCTACTTCGTCTTCG	GCTGTGGCATAGGCGTAGTT
*Slc48a1*	TGCTGGACCTCATCCTGTTT	TCCAGGTAGCCAGCATCTTC
*Tbx18*	AGCCCAAGACCAAGAGGACA	GTCGTCCTTGTCGTCCTTG
*Lrrn1*	GGAACCTGGTGCTGTTCTGT	GGCATCCAGTCTTCACAGGT
*ART1*	TCCTGGCTCTGCTCTTCATC	AGCCACAGTGTCCTTGCTCT
*Bdh1*	CAGCAAGGACCTGGTCAAGA	GCAGCCACAGTTGTCATTGA
*Nampt*	CAGTGTCCTGGTGAAGGTCA	GCTCCAGGTAGCCAAAGTCG
*Sirt1*	CAGCCCTTGTTGGAACATGA	TGGCAAGGGACTTGGTCTCT
*CACNA1C*	GGATCTGCATCTTCATCGGAA	TGGCACGATGTTGAAGATGCT
*β-actin*	ACTCCTATGTGGGTGACGAGG	CACACGCAGCTCATTGTAGAAG

### Non-targeted metabolomics and bioinformatic analysis

Metabolomic analysis was conducted on atrial tissues from the control, aged, and QPH groups. The supernatant was collected after centrifugation, and atrial tissues were homogenised after 80% methanol extraction of metabolites. Following machine protocols, all samples were processed by LC–MS. All initial chromatographic separations used a Thermo Scientific UltiMate 3000 HPLC. The reversed-phase separation was conducted using a Waters ACQUITY UPLC BEH C18 column (100mm × 2.1mm, 1.8 µm). The column oven was regulated at 35°C. The flow rate was 0.4 ml/min, with the mobile phase comprising solvent A (water, 0.1% formic acid) and solvent B (acetonitrile, 0.1% formic acid). Gradient elution conditions were as follows: 0–0.5 min, 5% B; 0.5–7 min, 5–100% B; 7–8 min, 100% B; 8–8.1 min, 100–5% B; 8.1–10 min, 5% B. The specific sample processing flow, chromatography, mass spectrometry conditions, and subsequent data analysis were performed as outlined in our previous publication [28].

The acquired MS data pretreatments including peak picking, peak grouping, retention time correction, second peak grouping, and annotation of isotopes and adducts was performed using XCMS software. The intensity of peak data was further preprocessed by metaX. Principal component analysis (PCA) was used to detect outliers and evaluate the effect of batch processing on the preprocessed data. Orthogonal partial least squares discriminant analysis (OPLS-DA) was performed by metaX to distinguish different variables among the groups, and the Variable Important for the Projection (VIP) value was calculated, with *P* < 0.05 and VIP value > 1 as the screening criteria for differential metabolites (DEMs).

### Integrating data from transcriptomics and metabolomics

Integrated transcriptomics and metabolomics analyses were conducted using MetaboAnalyst 5.0 (https://www.metaboanalyst.ca/) [29]. Gene and metabolite expression patterns were clustered into eight distinct trends with the OmicShare tool (https://www.omicsmart.com). Pearson correlation analysis was employed to associate trend-differentiated genes and metabolites, and the results were visualized using the Lianchuan BioCloud platform (https://www.omicstudio.cn/home) [30]. This comprehensive approach elucidated genetic and metabolic alterations, revealing potential mechanisms by which QPSM ameliorates aging-associated AF.

### The NAD^+^/NADH level of atrial tissue

According to the manufacturer's instructions, use the NAD^+^/NADH Assay Kit with WST-8 (Beyotime, China) to detect NAD^+^/NADH levels in atrial tissue. Weigh approximately 10–30 mg of atrial tissue, mince it, and add 200–600 μl of NAD^+^/NADH extraction solution at a ratio of 200 μl extraction solution per 10 mg of tissue. Homogenize at room temperature or on ice. Then centrifuge at 12,000 × g, 4 ℃ for 5–10 min, transfer 20 μL of the supernatant to a 96-well plate, incubate at 37 ºC for 10 min, and add 10 μL of the color reagent. Determine the NAD^+^/NADH ratio by measuring the absorbance of the mixture at 450 nm.

### Isolation of rat atrial myocytes and whole-cell patch-clamp technique

As previously demonstrated in investigations, atrial cells were acutely isolated through retrograde constant-rate (6 mL/min) perfusion via the aorta, utilizing the Langendorff apparatus [31]. In brief, the heart was quickly removed and mounted on a Langendorff apparatus. First, perfuse with calcium-containing buffer for 2 min to flush out blood residues. Then switch to Buffer A for 5 min until the heart completely stops beating. Next, perfuse with oxygen-saturated Buffer E. After approximately 30 min of perfusion, when the heart becomes soft, terminate digestion. Cut the left atrium and place it in KB solution, shred it with scissors, filter through a 100-mesh sieve, and transfer the cell suspension to a centrifuge tube. Centrifuge at 500 rpm for 30 s and discard the supernatant. Resuspend the cells in KB solution, let them settle naturally, and discard the supernatant. Use the gradient re-calcium method to re-calcium in three steps, discard the supernatant, add calcium-containing buffer, and set aside for use. The comprehensive methodology of Langendorff perfusion was elucidated in the supplementary methods (Supplementary File 2).

A whole cell patch clamp was used to measure the *I*_Ca,L_, while a current clamp was used for measuring a single cell action potential (AP) as described previously [32, 33]. In brief, use a micropipette puller (P-97, Sutter Instrument, USA) to pull glass electrodes with a resistance of 2.0–5.0Ω. Current signals were recorded with a MultiClamp 700B amplifier using the Digidata 1440A low noise data acquisition system (Axon Instruments). Data acquisition and command potential control were performed using pCLAMP 10.7 software (Axon Instruments). Record AP in current clamp mode and calculate action potential amplitude (APA), repolarization 50%, and 90% duration (APD50, APD90).

In voltage clamp mode, record the *I*_Ca,L_ current. Divide the current amplitude by the membrane capacitance to obtain current density. Plot the *I*_Ca,L_ current–voltage (I-V) curve in Origin 8.5 software using the clamped voltage as the X-axis and the current density as the Y-axis. To study the voltage dependence of channel activation and inactivation, plot the steady-state activation (SSA) and steady-state inactivation (SSI) curves, focusing on the half-activation voltage of the SSA and SSI curves. To investigate the rate at which channels recover from inactivation to reopening, plot the recovery from inactivation curve and calculate the recovery time constant. The preparation of intracellular and extracellular solutions was elucidated in the supplementary methods (Supplementary File 2).

### Mitochondrial superoxide assays

The isolated rat atrial cells were stained with MitoSOX (Molecular Probes) as described previously [34]. Add 1.0–2.0 mL of 5 μM MitoSOX reagent working solution to the cell-containing confocal dish. Incubate at 37 ℃ in the dark for 10 min. Afterwards, gently rinse the cells three times with pre-warmed HBSS buffer. Place under a confocal microscope, observe and photograph using an excitation wavelength of 396 nm and an emission wavelength of 610 nm.

### SA-β-galactosidase staining

According to the manufacturer's instructions, use the SA-β-galactosidase staining kit (Beyotime, China) to assess cell senescence. As previously described [35], isolated atrial myocytes were added to a six-well plate, and 500 μL of SA-β-gal staining fixative was added. After fixing at room temperature for 15 min, the fixative was discarded. Each group was then given 500 μL of working solution, and the six-well plate was incubated in a 37 °C incubator for 48 h. The senescence staining of the cells was observed using an optical inverted microscope at × 10 or × 20 magnification, and the proportion of senescent cells was counted by randomly selecting 5 fields of view.

### Histological analysis and immunohistochemistry

To ascertain the impact of QPSM on the morphology and structural remodeling of the AF rat atrium, a histopathological analysis of the left atrium was performed. The tissues were fixed via total immersion in 4% paraformaldehyde. Subsequently, paraffin-embedded tissues were serially sectioned at 5 μm thickness and stained with hematoxylin and eosin. Masson’s trichrome and Sirius red staining were employed to visualize collagen. For immunocytochemical staining, sections of the left atrium were incubated with Nampt (Thermo Fisher Scientific, PA1-1045). Images were captured using a Nikon Eclipse Ti-SR microscope, and the integrated optical density (IOD) along with the immunoreactive area were quantified using ImageJ software.

### Western blot analysis

The left atrial tissue was isolated and homogenized, centrifuged, and transferred. After blocking PVDT membranes with 5% skim milk powder in TBST for 1 h, the membranes were incubated with primary antibodies overnight at 4 °C. The primary antibodies used in this study include PARP1 (cat#ab191217), Nampt (cat#ab236874), Sirt1 (cat# ab110304), P21(cat# ab109520), P16 (cat# ab51243), CaV_1.2_ (cat# ab234438), SERCA2a (cat# ab 2861), Oxi-CaMKII (cat#. 07-1387), and RyR_2_^ser2814^ (cat#. PA5-104558). After incubation with secondary antibodies, the band intensity was observed using the ChemDoc gel imaging system and analyzed by Image J software.

### Statistical analysis

All results were statistically analysed using SPSS 26.0 and presented with GraphPad Prism 8.3 software. Measurement data adhering to a normal distribution were presented as mean ± standard deviation. One-way ANOVA was employed for overall group comparisons under the assumption of equal variance, whereas the least significant difference approach was utilised for pairwise comparisons between two groups. All statistical analyses utilised a two-sided test, with *P* < 0.05 deemed indicative of a statistically significant difference.

## Results

### QPSM reduced the susceptibility to AF in aging rat

We induced AF by administering burst stimulation and recorded the AF inducibility and duration as indicators. The results demonstrated that, compared to the Control group, rats in the Aged group were significantly more susceptible to AF (Fig. 1B, C). Both QPSM and Met reversed this susceptibility, resulting in a marked reduction in the AF induction rate (Fig. 1B, C). Likewise, the AF duration in the QPH, QPL, and Met groups was significantly shorter than in the Aged group (Fig. 1D). Additionally, simultaneous S1S2 programmed electrical stimulation was conducted to measure the atrial effective refractory period (AERP) at stimulation cycle lengths of 100ms and 120ms in each group. Compared to the Control group, AERP_100ms_ and AERP_120ms_ were shortened in the Aged group, although the differences were not statistically significant (Fig. 1F). In comparison to the Aged group, AERP_100ms_ and AERP_120ms_ were prolonged in the QPH, QPL, and Met groups, without statistically significant differences (Fig. 1F). These findings suggest that rats in the Aged group exhibit increased susceptibility to AF, and QPSM mitigates AF susceptibility and reduces AF duration.

### QPSM prevented atrial enlargement and fibrosis

**Fig. 2:**
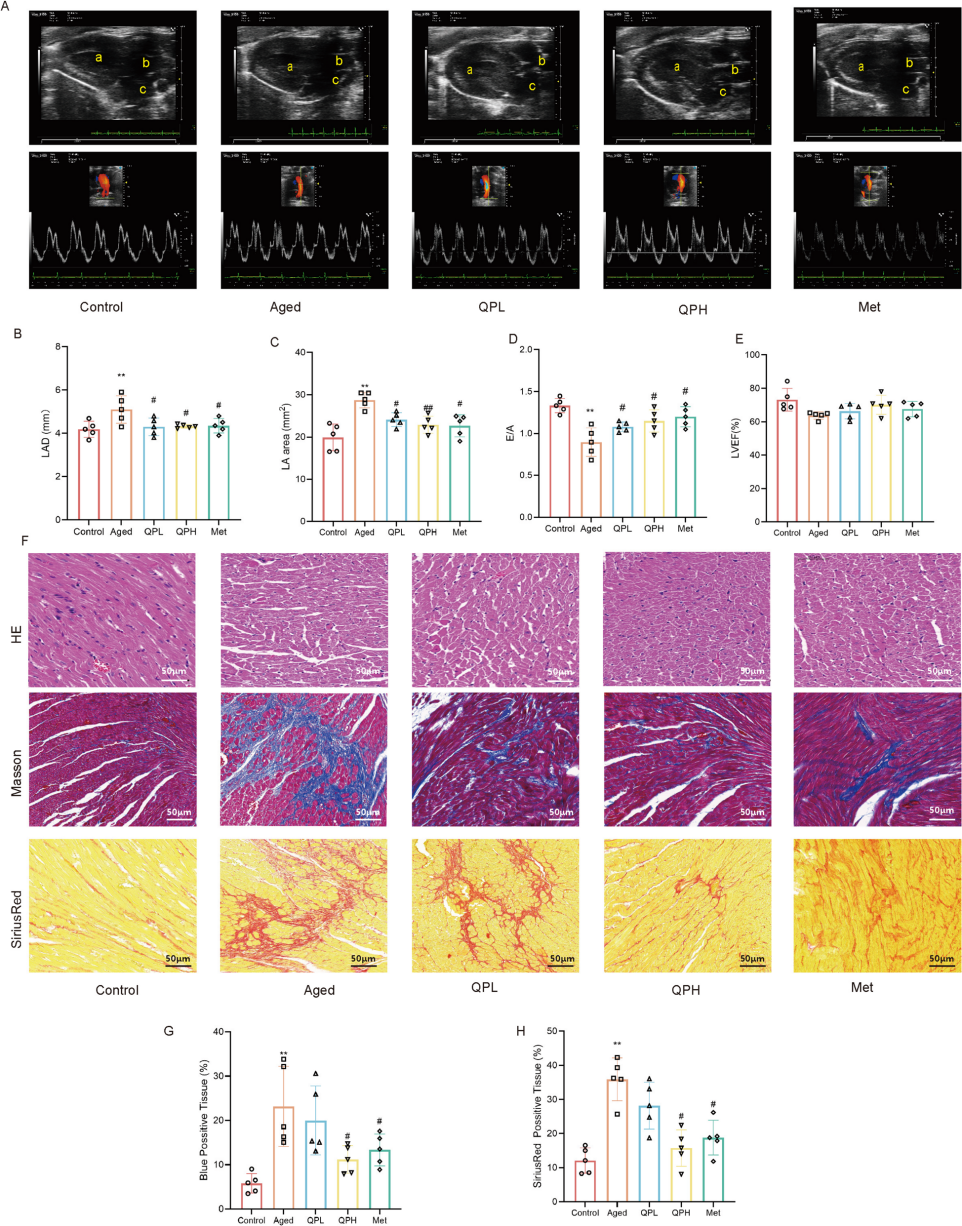
QPSM Prevented Atrial Enlargement and Fibrosis. **A** Representative echocardiographic imaging of the atria obtained during left ventricular end-systole from the four study groups. **B** LAD. **C** LA aera. **D** E/A. **E** LVEF. **F** Representative photomicrographs of H&E staining (× 200, 50μm). Representative photomicrographs of Masson staining (× 200, 50μm). Representative photomicrographs of SiriusRed staining (× 200, 50 μm). **G**, **H** Illustrates the quantitative ratio of the area of fibrosis to the area of the reference area. Each group included 6 rats. ^*^p < 0.05 and ^**^p < 0.01 compared with the control group, ^#^*p* < 0.05 and ^##^*p* < 0.01 compared with the Aged group

After the intervention, echocardiographic detection was performed on the rats in each group. The results showed that, compared with the Control group, the left atrial diameter (LAD) of the Aged group was significantly increased (Fig. 2A, B), and the left atrial area (LAA) was significantly enlarged (Fig. 2A, C), suggesting left atrial enlargement and atrial remodeling in the Aged rats. Furthermore, the E/A ratio demonstrated a notable decline (Fig. 2A, D), signifying impaired diastolic function in the aged subjects. Although the left ventricular ejection fraction (LVEF) exhibited a slight reduction, the discrepancy did not reach statistical significance (Fig. 2A, E). In comparison to the Aged group, the QPL, QPH, and Met group exhibited varying degrees of reduction in both LAD and LAA, as well as a concomitant rise in the E/A ratio, with these differences being statistically significant. Additionally, LVEF in the intervention group showed a minor improvement relative to the Aged group, although this enhancement failed to achieve statistical significance.

Under the microscope, HE-stained sections of atrial tissue from each group of rats were examined. The atrial tissue structure in the Control group appeared essentially normal, with intact myocardial cell architecture and occasional individual neutrophils observed in the stroma (Fig. 2F). In contrast, the Aged group exhibited marked disarray, with notable cell swelling. The myocardial cells in each medication group displayed a tendency towards normal arrangement, accompanied by reduced swelling. Further evaluation of atrial fibrosis was performed using Masson staining and Sirius Red staining. Compared to the Control group, the extent of atrial fibrosis in the Aged group rats was significantly exacerbated, with both Masson and Sirius Red staining indicating a substantial increase in the proportion of collagen fibers (Fig. 2F–H). Conversely, the proportion of collagen fibers in the total myocardial tissue area observed in each medication group was reduced to varying degrees, with the QPH and Met groups showing a statistically significant reduction in fibrosis (Fig. 2F–H).

### Exploring the targets involved in the protective effect of QPSM by transcriptomic analysis

**Fig. 3:**
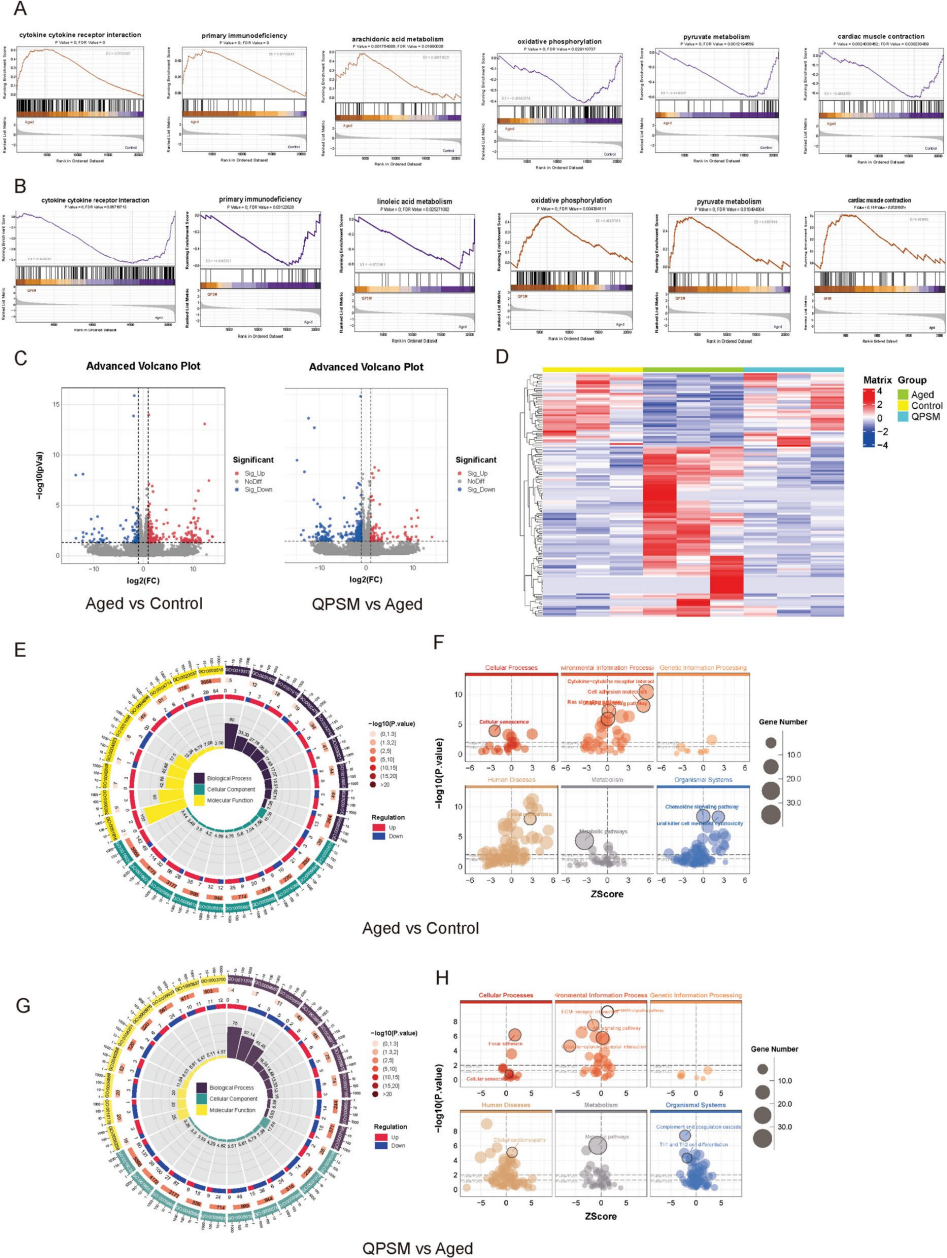
**A** KEGG enrichment analysis-based GSEA for the Aged vs Control groups. **B** KEGG enrichment analysis-based GSEA for the QPSM vs Aged groups. **C** Left: Volcano plot of differential genes for Aged vs Control, Right: Volcano plot of differential genes for QPSM vs Aged. In the plots, red dots indicate upregulated differential genes, blue dots indicate downregulated differential genes, and gray dots represent non-significant differential genes. **D** Heatmap of differential gene clustering among the Aged, Control, and QPSM groups. **E** GO annotation Loop Circos plot for Aged vs Control. **F** KEGG enrichment analysis Bubble Plot for Aged vs Control. **G** GO annotation Loop Circos plot for QPSM vs Aged. **H** KEGG enrichment analysis Bubble Plot for QPSM vs Aged

A total of 21,472 genes were identified in the current study. The GSEA analysis was performed to obtain a more comprehensive transcriptional profile by enriching gene expression across all samples globally, with the objective of identifying genes of significant biological relevance that are not differentially expressed [27, 36]. This technique clarifies the biological condition most similar to the expression pattern of a specific gene set [37]. As depicted in Fig. 3A, B and Supplementary File 3, the majority of gene sets exhibiting expression patterns aligned with the biological hallmarks of aging are predominantly associated with pathways such as inflammatory response, cytokine-cytokine receptor interaction, primary immunodeficiency, and arachidonic acid metabolism. In contrast, the gene sets in the QPSM group, whose expression patterns resemble normal biological states, are largely analogous and predominantly enriched in gene sets related to oxidative phosphorylation, pyruvate metabolism, and cardiac muscle contraction. These findings imply that QPSM may ameliorate D-Gal-induced atrial aging to some degree, potentially through the suppression of inflammation and the modulation of metabolic pathways.

Figure 3C, D illustrated that there were 577 DEGs between the Aged and Control groups, comprising 365 upregulated and 212 downregulated genes. Additionally, 531 DEGs in the QPSM group relative to the Aged group, with 155 genes being upregulated and 376 downregulated. Hierarchical clustering analysis elucidated the relative expression patterns of these differential genes across the three experimental groups. The findings indicated that the gene expression alterations induced by D-Gal were mitigated following QPSM treatment. The biological process analysis revealed that the differentially expressed genes in both comparisons were predominantly involved in immune response and ion transport processes (Figures E–H). The predominant cellular component terms pertained to membranes, the endoplasmic reticulum, and mitochondria, whereas the molecular function terms were predominantly associated with metal ion binding. Moreover, in alignment with the GSEA outcomes, the Kyoto Encyclopedia of Genes and Genomes (KEGG) pathway analysis highlighted that the DEGs were largely enriched in pathways related to inflammation and metabolic signaling, including Cytokine-cytokine receptor interaction, Chemokine signaling, Calcium signaling, Metabolic pathways, and Cellular senescence (Figures E–H).

### Exploring the targets involved in the protective effect of QPSM by metabolomics analysis

**Fig. 4:**
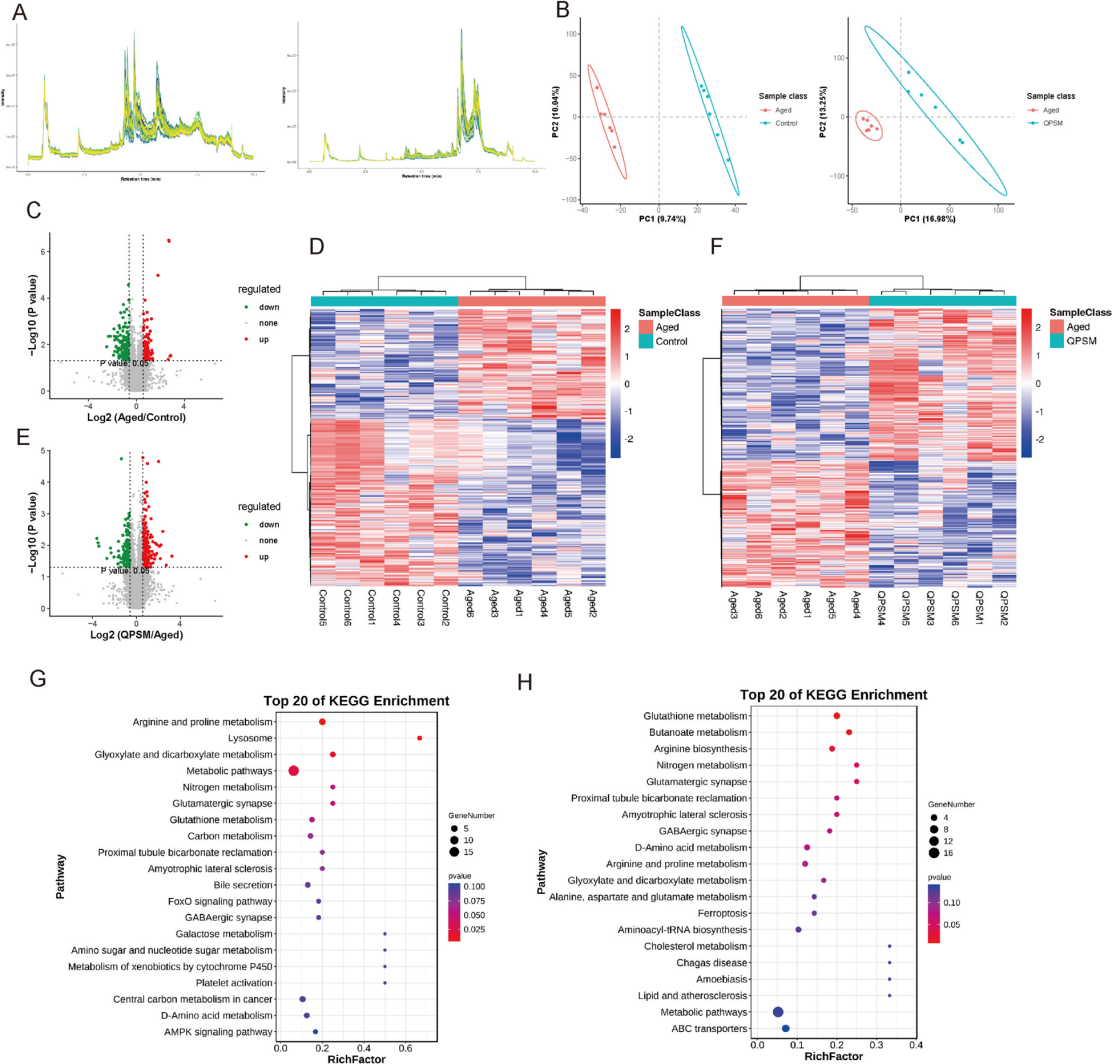
**A** Left: Positive ion TIC for each sample; Right: Negative ion TIC for each sample. **B** Left: OPLS-DA plot for Aged vs Control groups; Right: OPLS-DA plot for QPSM vs Aged groups. **C** Volcano plot of differential metabolic ions for Aged vs Control groups. **D** Heatmap of differential metabolic ions for Aged vs Control groups. **E** Volcano plot of differential metabolic ions for QPSM vs Aged groups. **F** Heatmap of differential metabolic ions for QPSM vs Aged groups. **G** KEGG bubble plot of differential metabolites for Aged vs Control groups. **H** Bubble plot of differential metabolites for QPSM vs Aged groups

A non-targeted metabolomics study was conducted in order to further investigate the mechanism by which QPSM enhances aging-related AF. Figure 4A displayed the total ion chromatograms of positive and negative ions for each sample. The retention times and peak areas of the various substances exhibit a significant overlap, suggesting a reliable result, good instrument stability, and a reasonable chromatographic gradient. The results of the PCA analysis demonstrated effective grouping of samples in both positive and negative ion modes. Furthermore, OPLS-DA (Fig. 4B) results revealed substantial changes in myocardial metabolic profiles among the Control, Aged, and QPSM groups. In the Aged group vs. Control group, 20,571 metabolic ions were obtained, with 137 upregulated and 208 downregulated, yielding 797 metabolites, 35 of which were DEMs, 19 in negative ion mode and 16 in positive ion mode (Fig. 4 C-D, Supplementary Material 3). In the QPSM group vs. Aged group, 20,571 metabolic ions were collected (Fig. 4E, F), with 242 upregulated and 201 downregulated, yielding 796 metabolites, including 50 DEMs, 31 in negative ion mode and 19 in positive ion mode. DEMs in the Aged group vs. Control group were mainly enriched in pathways such as Arginine and proline metabolism, Glyoxylate and dicarboxylate metabolism, Glutathione metabolism, FoxO signaling pathway, Galactose metabolism (Fig. 4G). DEMs in the QPSM group vs. Aged group were mainly enriched in pathways such as Glutathione metabolism, Nitrogen metabolism, Arginine and proline metabolism, Alanine, aspartate and glutamate metabolism, and Ferroptosis (Fig. 4H).

### Integrated transcriptomics and metabolomics unveil that QPSM mitigates aging-related AF susceptibility through the modulation of metabolic pathways

**Fig. 5:**
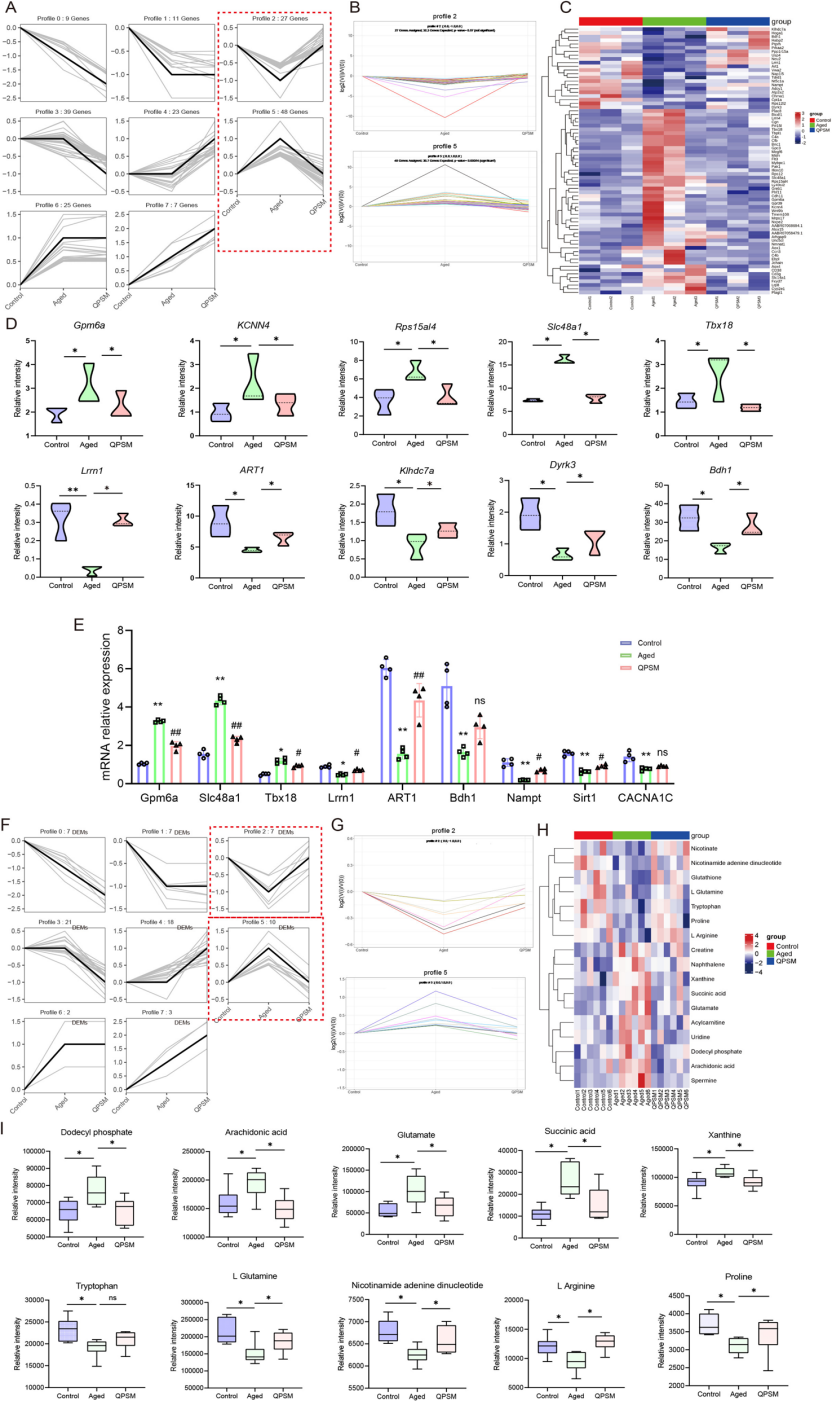
**A** Trend distribution of selected genes. **B** Distribution chart of down/up-regulated and up/down-regulated pattern genes. **C** Heatmap of trend gene distribution across groups. **D** Expression abundance of representative trend genes in each group. **E** Verification results of core genes in transcriptomics by RT-qPCR. **F** Trend distribution of selected metabolites. **G** Distribution chart of down/up-regulated and up/down-regulated pattern metabolites. **H** Heatmap of trend metabolite distribution across groups.** I** Expression abundance of representative trend metabolites in each group

In order to further clarify the effects of QPSM on aging-related AF left atrial genes and metabolites, one-way ANOVA was used to screen for genes and metabolites with differential expression among the three groups with *P* < 0.05 and perform trend analysis. As shown in Fig. 5A–D, 48 genes upregulated in the Aged group were downregulated by QPSM (up/down pattern), including: *Gpm6a, KCNN4, Rps15al4, Slc48a1, Tbx18*, etc.; 27 genes downregulated in the Aged group were upregulated by QPSM (down/up pattern), including *Lrrn1, ART1, Klhdc7a, Dyrk3, Bdh1,* etc. Based on RNA-seq analysis, we selected three genes exhibiting an up-down regulated pattern (*Gpm6a, Slc48a1, Tbx18*) and three genes exhibiting a down-up regulated pattern (*Lrrn1, ART1, Bdh1*) for further validation. Additionally, key pathway-associated genes (*Nampt, Sirt1, CACNA1C*) were included. RT-qPCR validation confirmed that the expression patterns of all selected genes were consistent with the RNA-seq results (Fig. 5E). At the same time, trend analysis of differentially expressed metabolites revealed that among the metabolites upregulated in the Aged group, 10 were downregulated by QPSM (up/down pattern), including Dodecyl phosphate, Arachidonic acid, Glutamate, Succinic acid, Xanthine, etc.; among the metabolites downregulated in the Aged group, 7 were upregulated by QPSM (down/up pattern), including Tryptophan, Spermine, Nicotinamide adenine dinucleotide, L Arginine, Proline, Glutathione, Nicotinate (Fig. F–I).

**Fig. 6:**
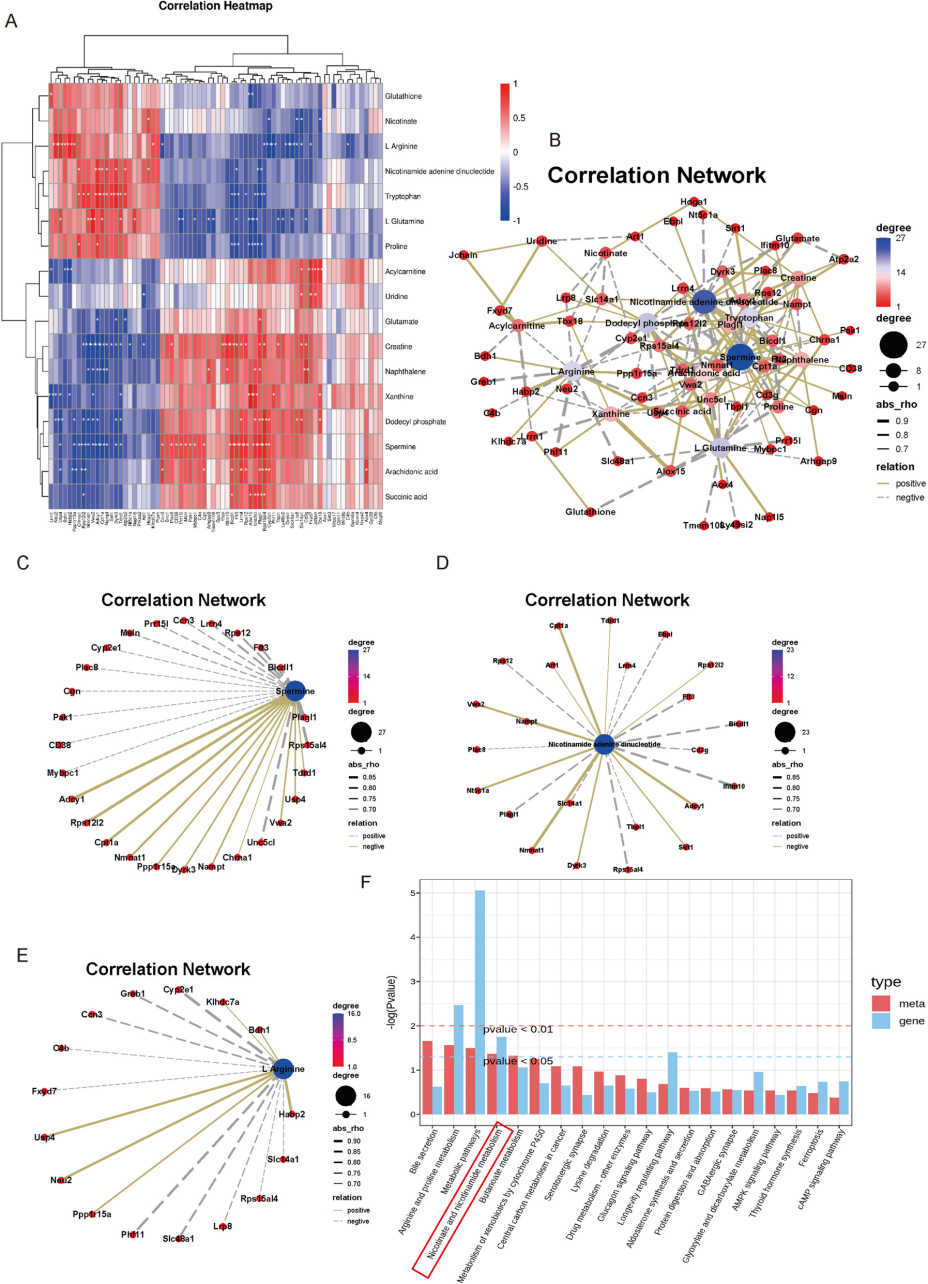
**A** Correlation heatmap trending gene and metabolite; **B** Correlation network map of trending genes and metabolites; **C** Spermine correlation network diagram; **D** L-arginine correlation network diagram; **E** Correlation network diagram of NAD; **F** Bar graph of KEGG co-enrichment of trending genes and metabolites

To explore the potential relationships between trending genes and metabolites, Pearson correlation analysis was used to create a heatmap to evaluate the correlations. As shown in Fig. 6A, each column represents a gene, and each row represents a metabolite. Red indicates a positive correlation between genes and metabolites, while blue indicates a negative correlation. To narrow the scope of the study, further screening was conducted on correlation data with |r|> 0.5 and P < 0.05 for the construction of a correlation network. The network diagram includes 56 nodes connected by 85 edges, with 45 pairs of nodes showing positive correlations and 49 pairs showing negative correlations. As shown in Fig. 6B, Spermine, L Arginine, Tryptophan, NAD, and L Glutamine were at the center of the network and associated with a large number of genes. Trending genes such as *Nmnat1*, *Cpt1a*, *Adcy1*, *Nt5c1a*, *Nampt*, and *Dyrk3* were correlated with the aforementioned trending metabolites (Figs. 6C–E). The KEGG co-enrichment furnace for trending genes and metabolites included Arginine and proline metabolism, Nicotinate and nicotinamide metabolism, Longevity regulating pathway, and several other metabolism pathways (Fig. 6F).

### QPSM reduces aging-related AF by regulating nampt expression and increasing NAD^+^ in atrial tissue

**Fig. 7:**
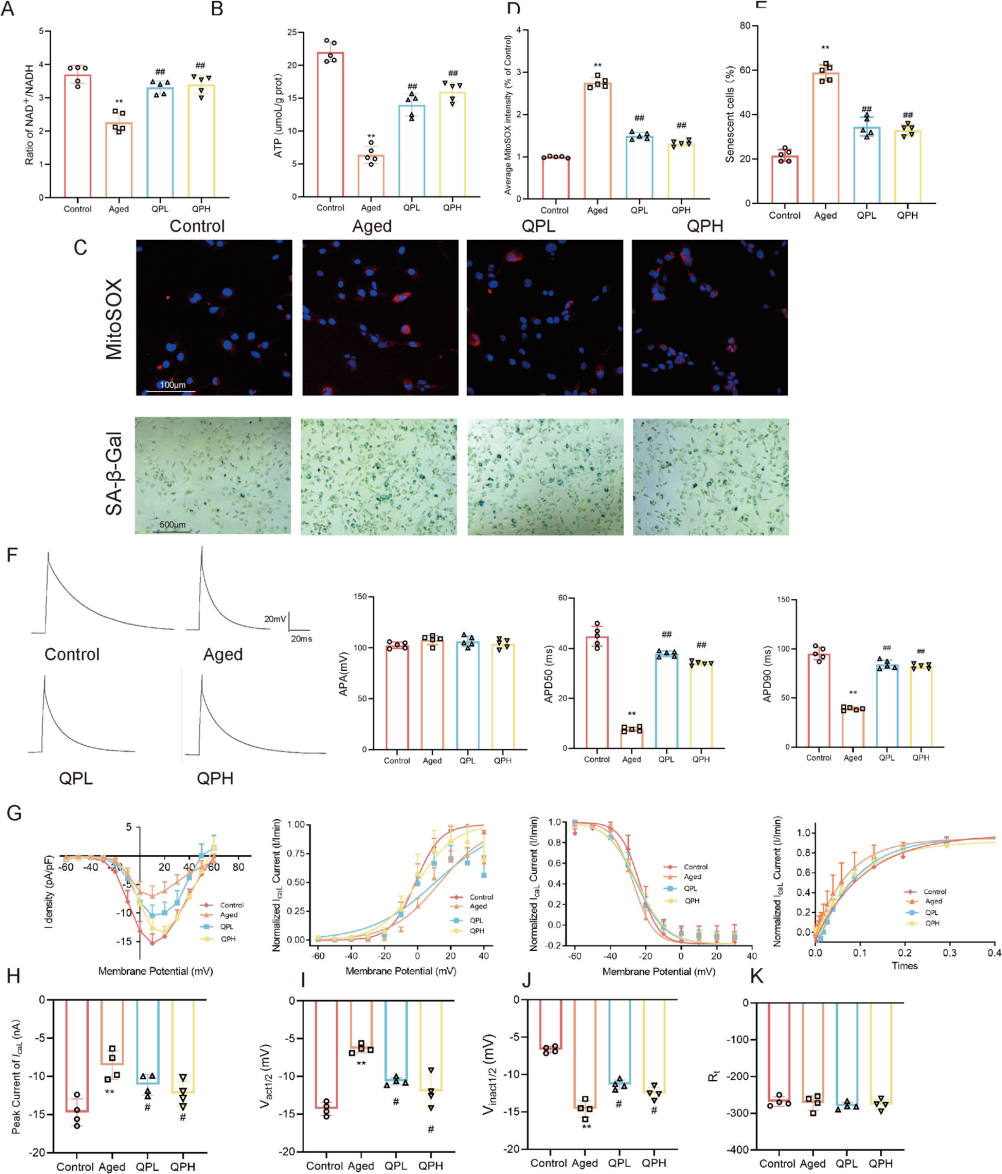
**A** NAD^+^/NADH levels in the left atrium of each group of rats; **B** ATP content in the left atrium of each group of rats; **C** Upper: Fluorescence intensity images of mitoSOX in atrial cells of each group of rats; Lower: SA-β-Gal staining results in atrial cells of each group of rats; **D** Statistical results of mitoSOX; **E** Statistical results of SA-β-Gal; **F** AP fitting curve and statistical results; **G** Ica L I-V curve, SSA curve, SSI curve, and recovery after inactivation of L-type calcium channels in atrial cells of each group of rats. **H** Peak current of *I*_ca L;_ **I** Vₐ_ct_₁/₂; **J** V_in_ₐ_ct_₁/₂; **K** R_t_

Based on the findings from the combined analysis and prior research, we propose that QPSM may mitigate age-relate AF by modulating Nampt expression and elevating NAD + levels in atrial tissue. To investigate this hypothesis, we first measured the NAD^+^ content in atrial tissues across different groups. As illustrated in Fig. 7A, the NAD^+^/NADH ratio was significantly reduced in the Aged group but notably increased in the QPL and QPH groups. NAD^+^ serves as a critical electron carrier in oxidative phosphorylation and plays a key role in ATP production. Subsequently, we assessed ATP content in the atrial tissues of each group. Compared to the Control group, ATP levels were markedly lower in the Aged group, whereas both the QPL and QPH groups demonstrated varying degrees of ATP restoration (Fig. 7B). Given the influence of NAD^+^ levels on cellular oxidative stress, we employed laser confocal microscopy to measure mitoSOX fluorescence intensity in each group. As shown in Fig. 7C, D, the Control group exhibited weak red fluorescence signals, indicating low mitoSOX expression. In contrast, the Aged group displayed strong red fluorescence, reflecting heightened mitoSOX expression and elevated oxidative stress. Drug intervention in the QPL and QPH groups significantly reduced red fluorescence intensity compared to the Aged group, suggesting a decrease in oxidative stress levels. To further evaluate cellular aging in atrial cells, isolated single cells were stained with SA-β-gal (Fig. 7C, E). In the Aged group, deep blue products accumulated around the nuclei, and the number of positively stained cells increased significantly, indicating pronounced cellular aging. Conversely, in the QPL and QPH groups, the accumulation of deep blue products and the proportion of positively stained cells were markedly reduced compared to the Aged group, highlighting the potential anti-aging effects of QPSM.

Electrophysiological remodeling, marked by alterations in action potentials, is believed to facilitate the persistence and progression of AF. Isolated single atrial cells were employed to record action potentials using the current-clamp technique. As depicted in Fig. 7F, there was no significant difference in the action potential amplitude (APA) between groups. Compared to the Control group, both APD50 and APD90 were significantly reduced in the Aged group; conversely, APD50 and APD90 were significantly prolonged in the QPL and QPH groups compared to the Aged group. In voltage-clamp mode, following the application of specific stimuli, *I*_ca L_ changes were recorded for each group (−60mV to + 40mV). Current density was calculated by dividing current amplitude by membrane capacitance, and plotting current density against stimulus voltage yielded *I*_caL_ I–V curves. The I–V curve for each group exhibited a typical "inverted bell-shaped" profile for *I*_caL_, with the peak current density around 10 mV (Fig. 7G, H). Compared to the Control group, the I–V curve of the Aged group shifted upward, whereas after QPSM administration, the curve shifted downward. This suggests that aging can lead to a reduction in *I*_ca L_ across cell membranes, with primary differences between groups occurring at −20 mV to 20 mV. Compared to the Control group, *I*_caL_ amplitude decreased in the Aged group; this decrease was reversed by QPL and QPH administration.

SSA and SSI curves represent the voltage dependence of channel activation and inactivation, respectively. Boltzmann fitting revealed a rightward shift in SSA from the Control to QPH, QPL, and Aged groups. In the Aged group, the half-activation voltage (Vₐ_ct_₁/₂) decreased compared to the Control group, while it increased in the QPL and QPH groups relative to the Aged group, indicating a slower calcium channel activation in the Aged group (Fig. 7I). Conversely, SSI exhibited a leftward shift from Control to Aged rats. The half-inactivation voltage (V_inact₁/₂_) significantly increased in the Aged group compared to the Control group (Fig. 7J). Additionally, no notable differences were observed in the recovery curves among the four groups following inactivation, and the recovery time constants remained unchanged (Fig. 7K).

### QPSM inhibits aging-related AF by regulating Nampt, increasing NAD^+^ levels, and reducing cytosolic calcium overload

**Fig. 8:**
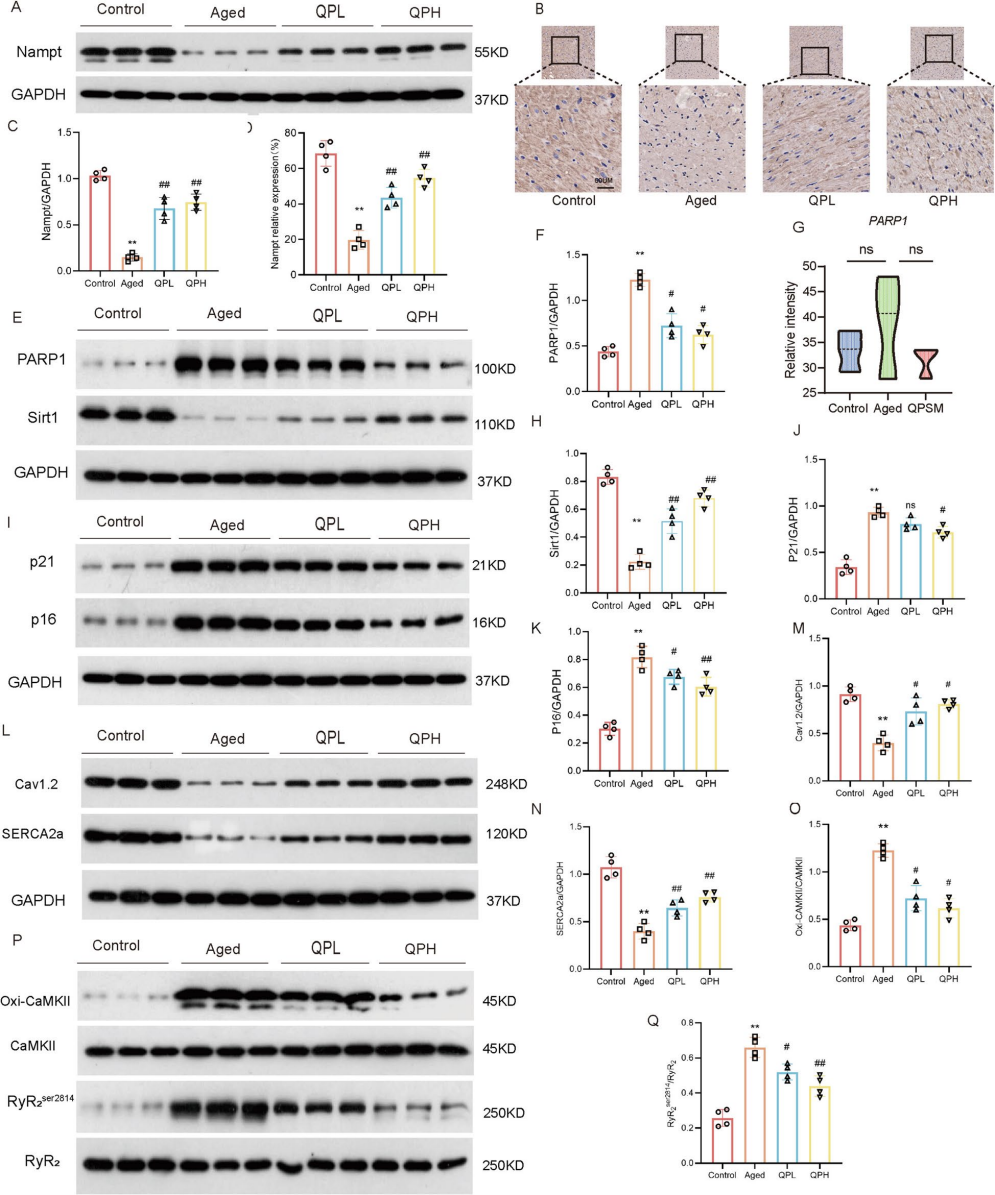
QPSM regulates Nampt expression, increases NAD^+^ levels, and inhibits cytosolic calcium overload. **A** Western blot bands of Nampt. **B** Representative photomicrographs of immunohistochemistry staining (× 200, 50 μm) in atrial tissues. **C** Nampt/GADPH. **D** Quantitative analysis of Namph relative expression by immunohistochemistry. **E** Western blot bands of PARP1 and SIRT1. **F** PARP1/GADPH. **G** PARP1 gene expression in the RNA-seq. **H** SIRT1/GADPH. **I**–**K** Western blot bands and quantitative analysis of p21 and p16. **L**–**N** Western blot bands and quantitative analysis of Cav1.2 and SERCA2a. **O**–**Q** Western blot bands and quantitative analysis of Oxi-CaMKII and RyR2^ser2814^

Nampt is a rate-limiting enzyme in the NAD^+^ salvage synthesis pathway and a key regulator of cellular NAD^+^. With combined omics analysis, this study assessed Nampt protein expression in atrial tissue. As demonstrated in Fig. 8A–D, the Aged group had much lower Nampt protein expression than the Control group, whereas the QPL and QPH groups had significantly higher levels. QPSM may improve NAD^+^ production by boosting Nampt expression, as IHC showed that Nampt was mostly in the cytoplasm of atrial cardiomyocytes and protein expression trends matched WB data.

Furthermore, NAD^+^ levels depend on both synthesis and consumption. Research shows that aging and AF enhance NAD^+^ consumption. Thus, this work further examined PARP1 and SIRT1 expression (Fig. 8E–H), critical NAD^+^ consumption pathway proteins. For the Aged group, SIRT1 expression was significantly reduced and PARP1 expression was significantly increased, while for the QPL and QPH groups, SIRT1 expression was significantly increased and PARP1 expression was significantly decreased. Transcriptomics analysis revealed that PARP1 gene expression was increased in the Aged group compared to the Control group and decreased in the QPSM group compared to the Aged group, although the difference was not statistically significant (Fig. 8G).

We also detected the expression of aging-related markers and calcium transport-related proteins. As demonstrated in Fig. 8I–K, the Aged group's left atrial tissue had considerably higher P21 and P16 protein expression than the Control group, indicating cellular senescence. The QPL and QPH groups had lower gene and protein expression levels of P53, P21, and P16 than the Aged group, with the QPH group being the most downregulation. This suggests that QPSM may slow rat atrial tissue aging. Additionally, the Aged group had lower Cav1.2 and SERCA2a protein expressions than the Control group, while the QPL and QPH groups had higher levels (Fig. 8L–N). Compared to the Control group, Oxi-CaMKII and RyR2^ser2814^ protein expressions were considerably enhanced in the Aged group and decreased in all treatment groups (Fig. 8O–Q).

## Discussion

It is well established that age is the most crucial determinant of AF risk, with recent studies highlighting the significant role of biological age [11, 38]. Existing literature reveals that aging-related AF is precipitated by various structural, electrophysiological, and molecular alterations associated with aging [2]. Progressive atrial conduction abnormalities and atrial fibrosis in aging individuals have been shown to influence AF through numerous clinical and experimental studies [39, 40]. In this research, we investigated the mechanism by which QPSM mitigates aging-related AF using a D-Gal-induced aging rat model. Our findings indicate that QPSM markedly reduces the inducibility and duration of AF in aged rats, ameliorates atrial electrical abnormalities, alleviates myocardial tissue fibrosis, and inhibits atrial remodeling. Transcriptomic and metabolomic analyses suggest that QPSM achieves these effects by inhibiting inflammation and regulating metabolism. Further experimental validation demonstrates that QPSM promotes NAD^+^ synthesis by upregulating Nampt, decreases NAD^+^ consumption by downregulating PARP1, alleviates oxidative stress, reduces Oxi-CaMKII and RyR_2_^ser2814^ expression to mitigate atrial cellular calcium overload, prolongs atrial myocyte APD, increases *I*_CaL_, suppresses myocardial cell electrical remodeling, and diminishes AF susceptibility.

The D-Gal-induced aging rat model is extensively utilized and recognized both domestically and internationally. By administering subcutaneous injections of D-Gal (at a dose of approximately 125–300 mg/kg per day) for 6–10 weeks, a stable aging model can be established [41–43] Specifically concerning cardiac aging, D-Gal induces severe structural disorganization of the heart, substantial interstitial spaces between cells, and an increase in apoptotic myocardial cells [44]. Based on this, our study further evaluated the efficacy of QPSM. The findings revealed that the incidence of AF in the QPSM group was significantly reduced, and correspondingly, the AFD was significantly shorter compared to the Aged group. However, there was no statistically significant difference in AERP among the groups. This may be attributed to the fact that the detection site for AERP in this study was the high left atrium, and other ectopic impulses, such as those from the pulmonary veins and right atrium, were not comprehensively evaluated [45–47]. Atrial structural remodeling is a hallmark change in AF myocardium, primarily involving atrial cavity dilation, myocardial cell hypertrophy, and interstitial fibrosis, which result in electrical conduction heterogeneity and conduction block. LAD is considered an independent predictor for AF occurrence [48]. The results of this study demonstrated that QPSM could reduce LAD in aged rats, decrease LA area, and ameliorate characteristic aging heart changes such as diastolic dysfunction and fibrosis. However, there was no significant difference in LVEF among the groups. It is noteworthy that clinical studies have also shown that in participants with normal LVEF, the E/A ratio decreases with age [49], which can serve as a marker for increased risk of heart failure with preserved ejection fraction [50].

Our prior investigations have demonstrated QPSM's capacity to restore intracellular calcium homeostasis in AF models through coordinated regulation of calcium transport machinery, including upregulation of *CACNA1C* and *SERCA2a* activity coupled with suppression of *CAMKII*-dependent signaling [20]. The current work extends these mechanistic observations to geriatric AF pathophysiology, demonstrating its capacity to orchestrate NAD⁺ metabolic rejuvenation via dual modulation of Nampt-mediated biosynthesis and PARP1-dependent catabolism. This pharmacodynamic synergy between calcium handling optimization unveils a novel therapeutic paradigm wherein QPSM concurrently addresses electromechanical and bioenergetic determinants of age-associated AF. Notably, the identified NAD⁺-sirtuin axis provides mechanistic rationale for its superior efficacy in senescent myocardium exhibiting characteristic mitochondrial ultrastructural derangements and progressive calcium handling incompetence.

NAD^+^ is a crucial coenzyme implicated in cellular aging and redox reactions, with its levels diminishing as various tissues age [51], including the heart [52]. NAD^+^ is considered a hallmark of cardiac aging [53]. Experimental AF models have confirmed that compounds that maintain NAD levels may represent an attractive treatment approach [54, 55]. Furthermore, the HF-AF ENERGY trial is a prospective intervention study, which is aims to investigate the cardioprotective effects of the NAD precursor nicotinamide riboside (NR) treatment in ischemic heart disease patients diagnosed with AF [56]. Nampt serves as the key rate-limiting enzyme in the salvage pathway and is a major determinant of intracellular NAD^+^ levels. Activators of Nampt have been proposed as therapeutic strategies to elevate tissue NAD^+^ levels. Nampt is abundantly expressed in cardiomyocytes, and recent studies have demonstrated its influence on various heart diseases, such as dilated cardiomyopathy, ischemia/reperfusion injury, and heart failure [57–59]. Feng D's research indicates that heterozygous Nampt knockout mice exhibit significantly increased atrial fibrillation susceptibility due to elevated diastolic calcium leaks [60]. In our study, QPSM enhances Nampt expression in atrial tissue and elevates NAD^+^ levels. Conversely, in the Aged group, reduced NAD^+^ content led to increased cardiomyocyte volume, heightened β-galactosidase activity, and upregulation of aging-related proteins P21 and P16, indicating cellular senescence. QPSM mitigated the proportion of senescent cells and the expression of P21 and P16, suggesting an amelioration of the senescent state. Concurrently, the reduction in NAD^+^ content induced oxidative stress damage in cells, and NADPH oxidase is a significant enzymatic source of ROS production in AF [61, 62]. ROS can enhance phosphorylation of RyR_2_ at the ser2814 site via oxidized CaMKII, promoting diastolic calcium leaks from the endoplasmic reticulum and triggering AF [60]. Consequently, we further investigated the expression of calcium transport-related proteins and genes. QPSM was found to upregulate SERCA2a expression, reduce CaMKII oxidation, and decrease RyR_2_ phosphorylation levels, suggesting that QPSM alleviates diastolic calcium overload in cardiomyocytes while augmenting SR calcium reserves, thereby reducing AF susceptibility.

The content of NAD^+^ is influenced by both its synthesis and consumption, and both aging and AF processes can elevate NAD^+^ consumption [63]. Consequently, we measured the expression of key proteins PARP1 and Sirt1 in the NAD^+^ consumption pathway. Results revealed that in the Aged group, Sirt1 expression significantly decreased, while PARP1 expression significantly increased. Research suggests that the pathological cycle mediated by PARP1 activation and NAD^+^ depletion plays a crucial role in maintaining genome integrity and cardiomyocyte function and serves as a significant regulatory mechanism for aging-related AF [64]. Oxidative DNA damage activating PARP1 is considered a pivotal factor leading to aging-related AF [65]. Based on these, using PARP inhibitors (such as ABT-888 or Olaparib) or NAD^+^ supplementation (in the form of Vitamin B3) can mitigate oxidative DNA damage and counteract structural remodeling and electrophysiological changes, thereby improving the contractile dysfunction of atrial cells [65, 66]. This indicates that pharmacological interventions inhibiting PARP1 may serve as practical and reliable treatment strategies for aging-related AF.

The electrophysiological characteristics of aging atrial cells include shortened APD and AERP, accompanied by intracellular calcium overload and decreased* I*_caL_ [67]. The reduction of *I*_caL_ further shortens APD and AERP by abbreviating the phase 2 plateau of the action potential, leading to DADs and triggered activities, ultimately inducing AF [68]. Electrical remodeling, characterized by changes in AP, is believed to contribute to the persistence and progression of AF. In this study, atrial myocytes in the Aged group demonstrated a decrease in *I*_caL_, along with a rightward shift in the SSA curve, a leftward shift in the SSI curve, and a depolarizing shift in the recovery curve post-inactivation. These alterations reflect a crucial mechanism of reduced *I*_caL_ gating kinetics in AF occurrence: slowed activation of L-type calcium channels on the cell membrane, accelerated inactivation, and delayed recovery post-inactivation [4, 69]. Additionally, echocardiography and pathological staining revealed that atria in the aged group exhibited hypertrophy, enlargement, collagen deposition, and fibrosis between myocardial cells, indicating structural remodeling, which can further elevate intracellular Ca^2+^ through various pathways, exacerbating atrial electrical remodeling and predisposing to AF [70, 71]. Moreover, intracellular calcium overload observed in this study can increase transient inward current, further triggering DAD and evolving into ectopic pacemakers in the atrium [72]. In summary, atrial myocytes in the aged group exhibit intracellular calcium overload and reduced *I*_caL_, leading to shortened or heterogeneous repolarization and heightened excitability of ectopic pacemakers, both significantly contributing to AF induction. QPSM can mitigate intracellular calcium overload and enhance *I*_caL_ by modifying the properties of L-type calcium channels in aging rats, thereby reducing AF susceptibility.

Over the past decade, considerable advancements have been achieved in the realm of senolytic research, yielding a profound comprehension of its potential therapeutic advantages. Therapeutic interventions involving senolytics, such as dasatinib, quercetin, miR-34a, and miR-570, have demonstrated promising results in the selective targeting of senescent cells, effectively mitigating aging processes, forestalling age-associated pathologies, fostering tissue rejuvenation, enhancing functional outcomes, and even exhibiting synergistic effects when combined with other treatments [73, 74]. In a groundbreaking study, Mehdizadeh and colleagues discovered that a validated senolytic cocktail, comprising dasatinib and quercetin, successfully inhibited biomarkers of cellular senescence, averted atrial fibrosis, and prevented the development of AF substrates in rats post-myocardial infarction [11]. This finding implies that manipulating cellular senescence could underpin novel therapeutic strategies for AF. Meanwhile, metformin has demonstrated promising anti-aging properties. A 2022 transcriptomic analysis identified it as a promising therapeutic candidate for AF [75]. Randomized controlled trials have yet to investigate the comprehensive effects of metformin on the prevalence of AF. However, empirical evidence indicates that metformin is associated with a reduced incidence of AF, with its cardioprotective benefits being distinct from its glucose-lowering properties [76, 77]. Our investigation employed metformin to assess its comparative pharmacodynamic effects with QPSM, revealing its capacity to mitigate age-related electrical and structural remodeling in AF. This aligns with various animal studies indicating that metformin can ameliorate left atrial structure and electrical remodeling by reducing intracellular reactive oxygen species, activating AMPK, stabilizing Ca^2+^ homeostasis and mitigating inflammation [78].

There are several limitations to our study: (1) The mechanism of QPSM was preliminarily verified in vivo by establishing an aging rat model, with no further in vitro experiments conducted for verification. In subsequent experiments, we will combine D-gal and rapid pacing stimulation to construct a rapid pacing-induced aging HL-1 cell model in vitro and further elucidate the mechanism of QPSM on aging-related AF by overexpressing or silencing target genes. (2) As a compound study, this research has only qualitatively identified the effective components of TCM granules without qualitative and quantitative analysis of the components in blood. Since the active components of TCM may not be the key components, future investigations will determine the plasma content of the active components and track their pharmacokinetic behavior to further elucidate the material basis and mechanism of QPSM. (3) Based on omics research, this study only selected NAD^+^ metabolism-related targets and pathways for preliminary verification of the mechanism of QPSM. In future experiments, the multi-target and multi-link characteristics of QPSM will be further delineated based on omics results.

## Conclusion

**Fig. 9:**
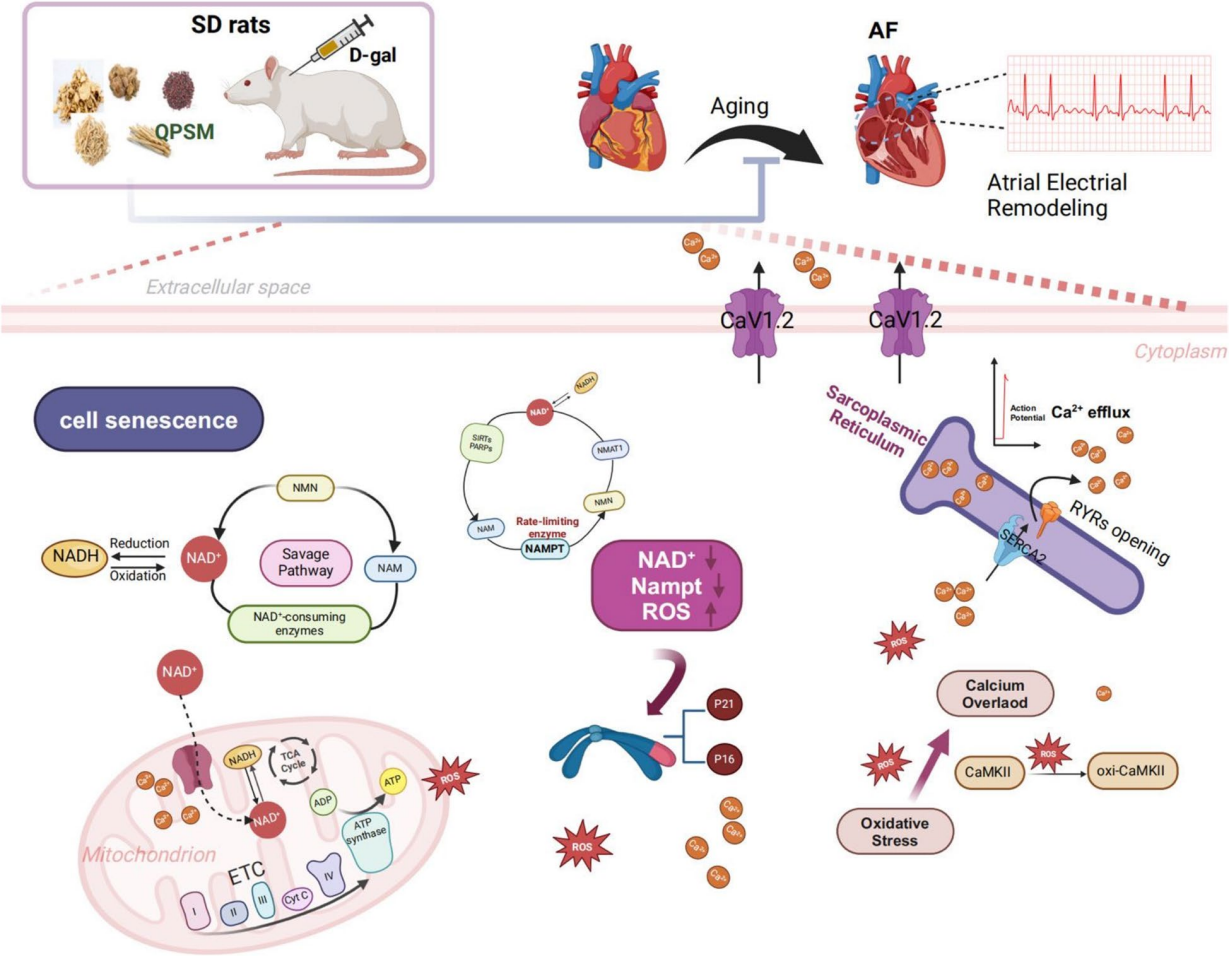
Diagram illustrating the mechanism through which QPSM diminishes susceptibility to AF in elderly rats

In summary, we found that QPSM might exert a therapeutic effect on aging-related AF by targeting calcium homeostasis via experimental verification in aged rats as shown in Fig. 9. These findings provide novel insights into the regulatory role of QPSM in the treatment of AF and hold promise for herb-based complementary and alternative therapy.

## Supplementary Information

Supplementary material 1.

Supplementary material 2.

Supplementary material 3.

## Data Availability

Data will be made available on request.

## Abbreviations

AF: Atrial fibrillation
QPSM: Qi-Po-Sheng-Mai Granule
AMPK: Adenosine monophosphate activated protein kinase
TCM: Traditional Chinese medicine
UHPLC: Ultra performance liquid chromatography
DEGs: Differentially expressed genes
FDR: False discovery rate
FC: Fold change
GO: Gene ontology
KEGG: Kyoto encyclopedia of genes and genomes
GSEA: Gene set enrichment analysis
PCA: Principal component analysis
OPLS-DA: Orthogonal partial least squares discriminant analysis
VIP: Variable important for the projection
DEMs: Differential metabolites
APA: Action potential amplitude
APD: Action potential duration
SSA: Steady-state activation
SSI: Steady-state inactivation
IOD: Integrated optical density
NAD: Nicotinamide adenine dinucleotide
Nampt: Nicotinamide phosphoribosyltransferase
PARP1: Poly-ADP-ribose polymerase 1
AERP: Atrial effective refractory period
LAD: Left atrial diameter
LAA: Left atrial area
LVEF: Left ventricular ejection fraction
CAMKII: Calcium/calmodulin-dependent protein kinase II
RyR2: Ryanodine receptor2
SR: Sarcoplasmic reticulum
