# Supplementary material for: Multi-omics reveals mechanism of Qi-Po-Sheng-Mai granule in reducing atrial fibrillation susceptibility in aged rats
Source: Chin Med. 2025 Sep 3;20:118. doi: 10.1186/s13020-025-01154-6 (PMC12406385; doi:10.1186/s13020-025-01154-6)
Supplement: Supplementary file 1 — Supplementary material 1. [file 13020_2025_1154_MOESM1_ESM.docx]

1. **QPSM Granule Sample Extraction**

The QPSM Granule sample is placed in a freeze-dryer for vacuum freeze-drying, and then ground using a grinder at a frequency of 30HZ for 1.5 minutes until the sample is in powder form. Weigh 100mg of the powder and dissolve it in 1.2mL of 70% methanol extraction solution, vortexing every 30 minutes for 30 seconds each time, for a total of 6 times. The sample is then stored in a refrigerator at 4℃ overnight. After centrifuging at 12000 rpm for 10 minutes, the supernatant is collected, filtered with a 0.22μm microporous membrane, and stored for subsequent analysis.

1. **Chromatography-Mass Spectrometry Acquisition Conditions**

Chromatographic separation was performed using a Waters HSS T3 (2.1×100mm, 1.8 μm) column. The column temperature was set at 35℃, the mobile phase A was 0.1% formic acid aqueous solution, and B was acetonitrile, with a flow rate of 0.3 mL/min, and gradient elution was applied. The detection wavelength was 300 nm, with an injection volume of 1 μL each time.

The gradient procedure is as follows：

| Time | A% | B% | Curve |
| --- | --- | --- | --- |
| Initial | 95 | 5 | Initial |
| 5 | 85 | 15 | 6 |
| 10 | 75 | 25 | 6 |
| 15 | 68 | 32 | 6 |
| 20 | 60 | 40 | 6 |
| 25 | 95 | 5 | 6 |
| 30 | 95 | 5 | 6 |

1. **QPSM Granule** **Ingredient Identification Results**

QPSM Granule UPLC-UV chromatogram and UPLC-QTOF-MS total ion current map (positive and negative modes), as shown in Figure 1. The chromatographic peak substances of number 1-24 identified in the figure were well separated and detected. The sources of the 25 peaks were determined by comparing the UV profiles of the QPSM Granule prescription with those of each individual drug (FIG. 2).


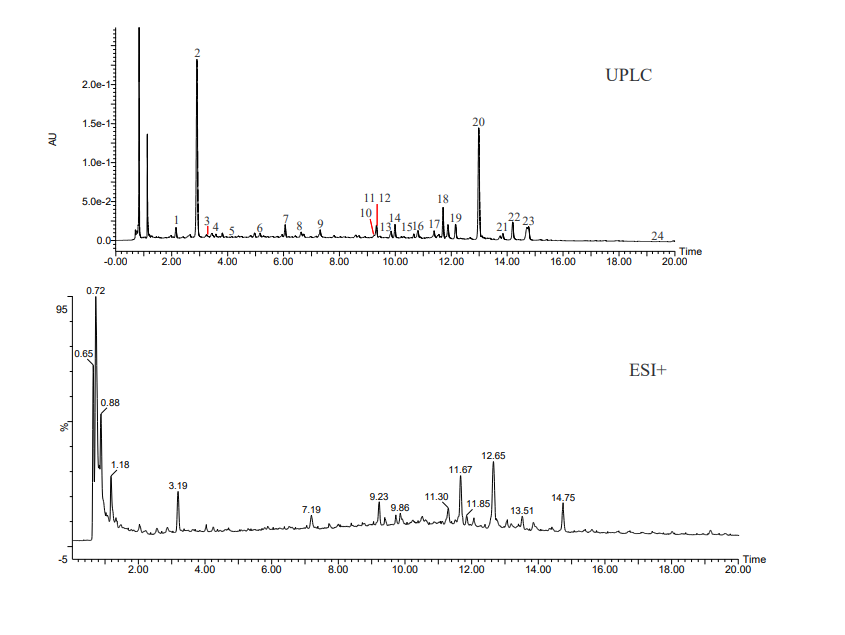


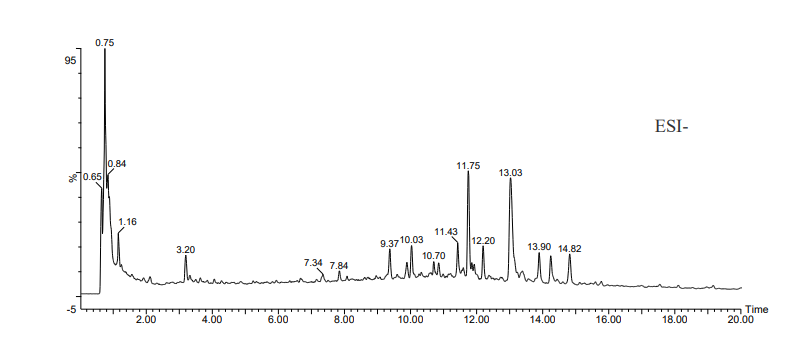


Fig1. QPSM Granule UPLC-UV chromatogram and UPLC-QTOF-MS total ion current


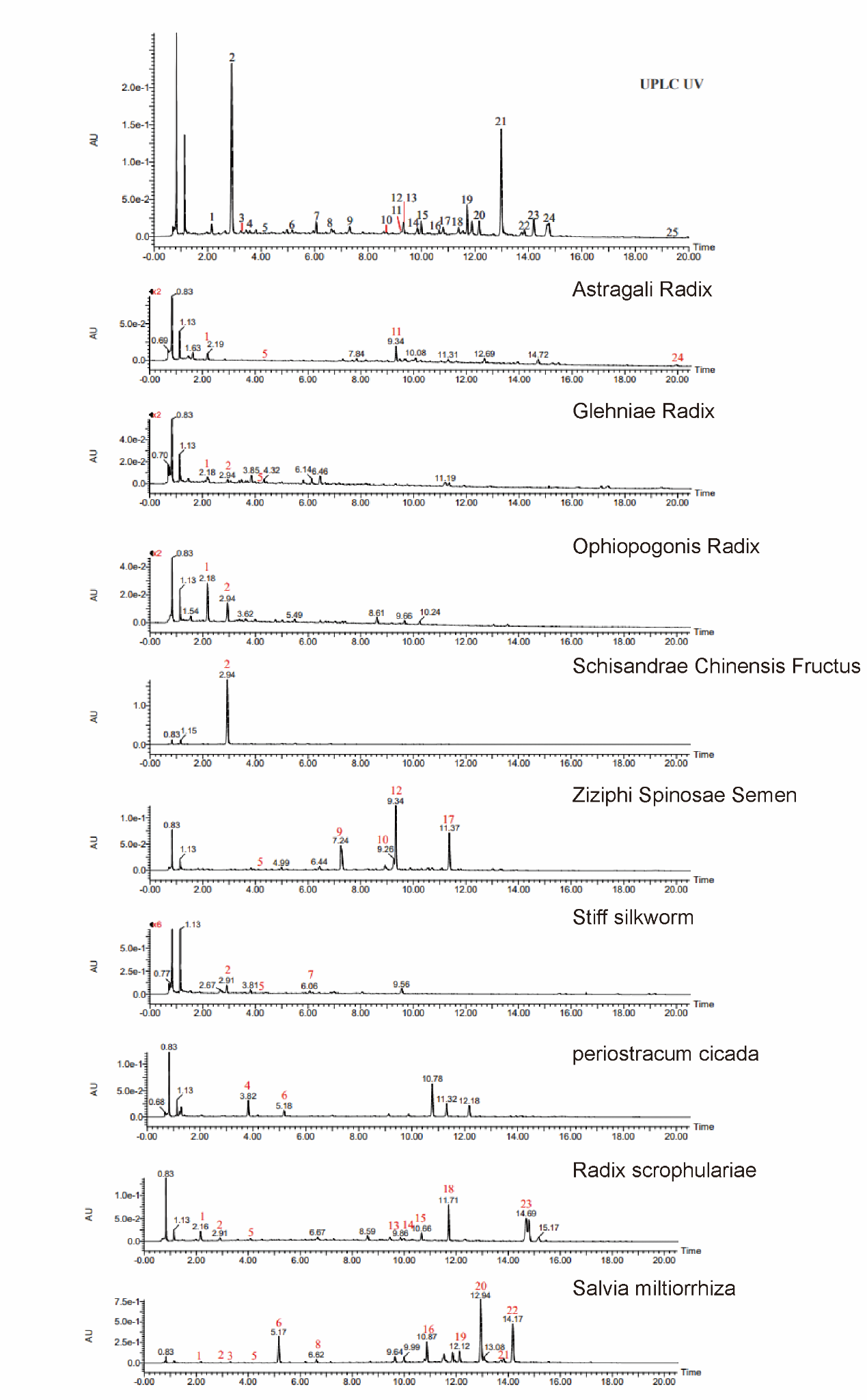


Fig 2. Comparison of UPLC-UV between QPSM granule prescription and each single herb
