# Supplementary material for: Multi-omics reveals mechanism of Qi-Po-Sheng-Mai granule in reducing atrial fibrillation susceptibility in aged rats
Source: Chin Med. 2025 Sep 3;20:118. doi: 10.1186/s13020-025-01154-6 (PMC12406385; doi:10.1186/s13020-025-01154-6)
Supplement: Supplementary file 2 — Supplementary material 2. [file 13020_2025_1154_MOESM2_ESM.docx]

**1.Electrophysiological analysis**

After 5 weeks of intragastric administration in each group of rats, atrial electrophysiological testing and AF induction were performed, and rat ECG signals were recorded using Power Lab and LabChart 7. After anesthesia with 2% pentobarbital (0.3 ml/100 g), electrodes were implanted in the subcutaneous tissue of the limbs, and limb lead II was used to collect surface ECG parameters for each group. After tracheal intubation and connection to a ventilator for assisted respiration, the chest cavity was opened at the third rib on the left side, and the pacing electrode was connected to the free wall of the left atrium. The detection content included: ① Measurement of AERP using S1S2 program stimulation. ② Induction of AF using atrial burst stimulation, recording AF induction rate and AF duration. AF is defined as >1s of irregular atrial electrograms (>800 bpm) accompanied by an irregular ventricular rate. AF duration is defined as the total duration of all AF episodes within 60 seconds for each rat. AF induction rate is defined as the number of AF occurrences/total burst pacing times × 100%. ③ Measurement of the maximum P-wave duration, P-wave dispersion, and PR interval in lead II. Analysis was performed using EMapScope 4.0 software. The measurement time for each rat was approximately 20 minutes.

**2.Echocardiography**

Five weeks after administration, echocardiographic monitoring was performed using the Vevo 3000 small animal ultrasound system. After hair removal, anesthesia was induced with 3% isoflurane and maintained at a lower dose (1.5%–2%), followed by fixation on the examination table. Echocardiographic parameters were obtained in the parasternal long-axis view. Two-dimensional echocardiography was used to measure the anterior-posterior diameter of the left atrium, the end-diastolic inner diameter of the left ventricle, and the end-systolic inner diameter of the left ventricle, with the left atrial area traced, and five cardiac cycles were continuously measured. All left atrial measurements were made with a caliper and only measured when the ventricular systole and the aortic cusp tissue were clearly visible. The average value was used to define each measurement point for each instance. Left ventricular ejection fraction was calculated using the following formula:

LVEDV (left ventricular end − diastolic volume) = [7/ (2.4 + LVEDD)] × LVEDD

LVESV (left ventricular end − systolic volume) = [7/ (2.4 + LVESD)] × LVESD

LVEF = [(LVEDV − LVESV)/LVEDV] × 100%

**3. Langendorff perfusion**

3.1 Solution and Apparatus Preparation

Oxygenate the desktop solution and KB solution for 30 minutes. Filter the desktop solution through a 0.22 μm microporous membrane. Buffer A: Take 100 mL of the aforementioned desktop solution, add 125.1 mg Taurine and 10 μL of 100 mmol/L CaCl2. Buffer E: Take 40 mL of Buffer A, add 30 mg Collagenase II. Use ultrapure water to clean the entire perfusion apparatus. Simultaneously turn on the thermostat to maintain the outflow temperature at 36.5-36.8℃. Open the gas cylinder and continue to oxygenate Buffer A and Buffer E. Flush the perfusion apparatus with calcium-containing desktop solution, expel all air bubbles, and fill the entire apparatus.

3.2 Isolation of Atrial Myocytes

Weigh the rats from each group, anesthetize them with an intraperitoneal injection of 2% sodium pentobarbital (0.33 mL/100 g). Open the chest to extract the heart, place it in pre-cooled 4°C calcium-containing Tyrode solution, remove excess tissue, and locate the aorta. Ligate the aorta at the base of the cannula. Note: The time from opening the chest to starting perfusion should not exceed 2 minutes. Perform perfusion at a constant flow rate of 6 mL/min. First, use calcium-containing Tyrode solution to perfuse for 2 minutes to flush out blood. Then switch to Buffer A for 5 minutes until the heart completely stops beating. Subsequently, use oxygen-saturated Buffer E for continuous perfusion. After approximately 30 minutes, when the heart becomes soft, terminate digestion. Use a syringe to draw 10 mL of Buffer A to wash out the digestive enzymes from the heart. Cut the left atrium and place it in KB solution. Mince the tissue with scissors, gently pipette to accelerate cell dissociation, and filter through a 100-mesh sieve. Transfer the cell suspension into a centrifuge tube, centrifuge at 500 rpm for 30 seconds, and discard the supernatant. Resuspend the cells in KB solution, let them stand for natural sedimentation for 6 minutes, and discard the supernatant. Reintroduce calcium. Perform a gradient calcium reintroduction in three steps. Finally, discard the supernatant, add calcium-containing Tyrode solution, and let it stand for further use.

**4. The preparation of intracellular and extracellular solutions**

*I*_ca,L_ extracellular fluid

| Reagent | Molecular weight（g/mol) | Concentration | Weight(g/100ml) |
| --- | --- | --- | --- |
| NaCl | 58.44 | 140 | 0.8182 |
| KCl | 74.55 | 4 | 0.0298 |
| CaCl_2_ | 110.98 | 2 | 0.0222 |
| MgCl_2_ | 95.21 | 1 | 0.0095 |
| HEPES | 238.3 | 10 | 0.2383 |
| Glucose | 180.16 | 10 | 0.1802 |

The PH was adjusted to 7.4 using CsOH.

*I*_ca,L_ electrode internal fluid

| Reagent | Molecular weight（g/mol) | Concentration | Weight(g/100ml) |
| --- | --- | --- | --- |
| CsCl | 168.36 | 120 | 2.0203 |
| CaCl_2_ | 110.98 | 1 | 0.0111 |
| MgCl_2_ | 95.21 | 5 | 0.0476 |
| HEPES | 238.3 | 10 | 0.2383 |
| EGTA | 380.35 | 11 | 0.4184 |
| Na_2_ATP | 605.24 | 5 | 0.3026 |

The PH was adjusted to 7.2 using CsOH.

AP electrode internal fluid

| Reagent | Molecular weight（g/mol) | Concentration | Weight(g/100ml) |
| --- | --- | --- | --- |
| NaCl | 58.44 | 140 | 0.8182 |
| KCl | 74.55 | 4 | 0.0298 |
| CaCl2 | 110.98 | 1 | 0.0111 |
| MgCl2 | 95.21 | 1 | 0.0095 |
| HEPES | 238.3 | 10 | 0.2383 |
| Glucose | 180.16 | 5 | 0.0901 |

The PH was adjusted to 7.36 using CsOH.

AP electrode internal fluid

| Reagent | Molecular weight（g/mol) | Concentration | Weight(g/100ml) |
| --- | --- | --- | --- |
| K-aspartate | 171.19 | 120 | 2.0543 |
| KCl | 74.55 | 20 | 0.1491 |
| MgCl2 | 95.21 | 1 | 0.0095 |
| HEPES | 238.3 | 10 | 0.2383 |
| Glucose | 180.16 | 10 | 0.1802 |
| Na_2_ATP | 605.24 | 4 | 0.2421 |

The PH was adjusted to 7.3 using CsOH.
