# Supplementary material for: Multi-omics reveals mechanism of Qi-Po-Sheng-Mai granule in reducing atrial fibrillation susceptibility in aged rats
Source: Chin Med. 2025 Sep 3;20:118. doi: 10.1186/s13020-025-01154-6 (PMC12406385; doi:10.1186/s13020-025-01154-6)
Supplement: Supplementary file 3 — Supplementary material 3. [file 13020_2025_1154_MOESM3_ESM.docx]

**Table 1. GSEA based on KEGG enrichment analysis in Aged group vs Control group**

| **NAME** | **SIZE** | **ES** | **NES** | ***P*** | **FDR** |
| --- | --- | --- | --- | --- | --- |
| Cytokine receptor interactions | 228 | 0.5153 | 2.1742 | 0 | 0 |
| Immunodeficiency | 32 | 0.6772 | 2.0847 | 0 | 0 |
| Th1 and Th2 cells differentiate | 78 | 0.5214 | 1.8875 | 0 | 0.0012 |
| Natural killer cell-mediated cytotoxicity | 85 | 0.5063 | 1.8734 | 0 | 0.0016 |
| T-cell receptor signaling pathways | 96 | 0.4797 | 1.7932 | 0 | 0.0053 |
| Antigen processing and presentation | 70 | 0.5018 | 1.7734 | 0 | 0.0065 |
| Th17 cell differentiation | 93 | 0.4627 | 1.7416 | 0 | 0.0087 |
| NFκB signaling pathway | 96 | 0.4504 | 1.7052 | 0 | 0.0122 |
| Arachidonic acid metabolism | 51 | 0.4891 | 1.6601 | 0.0017 | 0.0198 |
| Metabolic effects of cytochrome p450 on exogenous drugs | 49 | 0.4788 | 1.6263 | 0.0017 | 0.0246 |
| Metabolism of carbon | 111 | -0.5603 | -2.2765 | 0 | 0 |
| Citric Acid Cycle (TCA cycle) | 29 | -0.7009 | -2.2522 | 0 | 0 |
| Metabolism of propionate | 34 | -0.6626 | -2.1503 | 0 | 0.0004 |
| Pyruvate metabolism | 37 | -0.6043 | -2.0191 | 0 | 0.0012 |
| Ubiquitin-mediated proteolysis | 131 | -0.4273 | -1.8013 | 0 | 0.0143 |
| Pathways for regulating longevity in multiple species | 59 | -0.4678 | -1.7392 | 0.0024 | 0.0221 |
| Oxidative phosphorylation | 119 | -0.4166 | -1.7122 | 0 | 0.0291 |
| Contraction of myocardium | 74 | -0.4494 | -1.7000 | 0.0024 | 0.030 |
| Adrenergic signaling in cardiomyocytes | 134 | -0.3912 | -1.6419 | 0 | 0.0468 |
| AMPK Signaling Pathway | 118 | -0.3484 | -1.4341 | 0.0081 | 0.1527 |

**Table 2 GSEA analysis based on KEGG enrichment in QPSM group vs Aged group**

| **NAME** | **SIZE** | **ES** | **NES** | ***P*** | **FDR** |
| --- | --- | --- | --- | --- | --- |
| Linoleic acid metabolism | 24 | -0.6731 | -1.8659 | 0 | 0.0252 |
| Primary immunodeficiency | 32 | -0.6043 | -1.7713 | 0 | 0.0312 |
| Cytokine receptor interactions | 228 | -0.4343 | -1.6602 | 0 | 0.0671 |
| Natural killer cell-mediated cytotoxicity | 85 | -0.4559 | -1.5947 | 0.0024 | 0.0940 |
| Arachidonic acid metabolism | 51 | -0.4834 | -1.5222 | 0.0215 | 0.1363 |
| Metabolism of propionate | 34 | 0.6329 | 2.2163 | 0 | 0.0019 |
| Metabolism of carbon | 111 | 0.4932 | 2.1105 | 0 | 0.0031 |
| Citric Acid Cycle (TCA cycle) | 29 | 0.6295 | 2.0802 | 0 | 0.0022 |
| Oxidative phosphorylation | 119 | 0.4623 | 1.9924 | 0 | 0.0043 |
| Pyruvate metabolism | 37 | 0.5597 | 1.91295 | 0 | 0.0104 |
| Contraction of myocardium | 74 | 0.4139 | 1.6457 | 0 | 0.0728 |
| FOXO signaling pathway | 127 | 0.3753 | 1.6405 | 0 | 0.0699 |
| Protein processing in the endoplasmic reticulum | 165 | 0.3679 | 1.6379 | 0 | 0.0666 |
| Longevity Regulation Pathways | 85 | 0.4033 | 1.6355 | 0 | 0.0602 |
| Methyl butyrate metabolism | 26 | 0.4842 | 1.5259 | 0.0422 | 0.1102 |

**Table 3 Differential metabolites in Aged vs Control group**

| **NO.** | **ID** | **MZ** | **RT** | **Metabolite** | **Ratio** | ***P*** | **VIP** | **Kegg ID** |
| --- | --- | --- | --- | --- | --- | --- | --- | --- |
| 1 | neg | 127.05 | 1.15 | Naphthalene | 0.73 | 0.02 | 2.18 | C00829 |
| 2 | neg | 133.01 | 9.19 | D-(+)-Malic acid | 0.71 | 0.02 | 1.86 | C00497 |
| 3 | neg | 145.06 | 1.15 | L-Glutamine | 0.70 | 0.01 | 2.39 | C00064 |
| 4 | neg | 149.06 | 3.20 | 2-Methoxy-4-vinylphenol | 0.65 | 0.04 | 2.33 | C17883 |
| 5 | neg | 151.03 | 1.46 | Xanthine | 1.17 | 0.03 | 1.19 | C00385 |
| 6 | neg | 181.07 | 0.77 | Dulcitol | 6.62 | 0.00 | 7.30 | C01697 |
| 7 | neg | 184.00 | 1.09 | Phosphoserine | 0.77 | 0.03 | 1.56 | NA |
| 8 | neg | 192.05 | 2.83 | L-Glutamic acid | 6.98 | 0.03 | 4.04 | C00025 |
| 9 | neg | 265.15 | 3.73 | Dodecyl phosphate | 1.19 | 0.03 | 1.27 | NA |
| 10 | neg | 338.99 | 0.65 | Fructose 1,6-bisphosphate | 0.35 | 0.02 | 3.84 | C00354 |
| 11 | neg | 338.99 | 9.12 | alpha-D-Glucose-1,6-diphosphate | 0.71 | 0.00 | 2.13 | C01231 |
| 12 | neg | 369.00 | 0.65 | D-Ribulose 1,5-bisphosphate | 0.47 | 0.03 | 3.31 | C01182 |
| 13 | neg | 378.10 | 1.24 | S-Lactoylglutathione | 0.67 | 0.04 | 1.74 | C03451 |
| 14 | neg | 426.02 | 0.84 | ADP | 0.69 | 0.02 | 1.89 | C00008 |
| 15 | neg | 582.38 | 5.51 | LysoPC 18:0 | 1.21 | 0.04 | 1.22 | C04230 |
| 16 | neg | 585.49 | 7.50 | FAHFA 38:4; FAHFA(20:4/18:0) | 1.88 | 0.01 | 2.69 | - |
| 17 | neg | 804.61 | 4.97 | Plasmenyl-PC 35:0 | 0.78 | 0.03 | 1.22 | C00958 |
| 18 | neg | 824.58 | 6.61 | Plasmenyl-PC 36:4 | 2.41 | 0.00 | 4.11 | C00958 |
| 19 | neg | 834.53 | 4.75 | 2-Docosahexaenoyl-1-stearoyl-sn-glycero-3-phosphoserine | 1.19 | 0.04 | 1.20 | NA |
| 20 | pos | 116.07 | 1.25 | Proline | 0.84 | 0.00 | 1.15 | C00148 |
| 21 | pos | 146.16 | 2.24 | Spermidine | 0.81 | 0.05 | 1.37 | C00315 |
| 22 | pos | 154.06 | 1.21 | Creatine | 1.28 | 0.00 | 1.88 | C00300 |
| 23 | pos | 184.09 | 0.78 | L-Carnitine | 1.37 | 0.00 | 2.25 | C00318 |
| 24 | pos | 184.09 | 1.17 | Ginkgotoxin | 1.35 | 0.04 | 1.99 | NA |
| 25 | pos | 203.22 | 2.26 | Spermine | 1.34 | 0.00 | 2.11 | C00750 |
| 26 | pos | 245.08 | 1.62 | Uridine | 1.34 | 0.05 | 1.79 | C00299 |
| 27 | pos | 248.15 | 1.59 | 12-Cytisineacetamide | 0.49 | 0.02 | 2.49 | NA |
| 28 | pos | 278.06 | 1.05 | Mannose 6-phosphate | 1.29 | 0.05 | 1.21 | C00275 |
| 29 | pos | 341.00 | 0.65 | D-myo-Inositol-1,5-diphosphate | 0.28 | 0.00 | 4.49 | NA |
| 30 | pos | 341.00 | 0.97 | D-myo-Inositol-4,5-diphosphate | 0.46 | 0.01 | 3.33 | NA |
| 31 | pos | 353.00 | 0.65 | Furosemide | 0.50 | 0.03 | 3.37 | C07017 |
| 32 | pos | 358.03 | 0.66 | D-myo-Inositol-1,5-diphosphate | 0.40 | 0.00 | 3.46 | NA |
| 33 | pos | 442.35 | 4.48 | 3-Hydroxyoleylcarnitine | 0.67 | 0.04 | 2.07 | NA |
| 34 | pos | 448.34 | 4.84 | Acylcarnitine 20:4 | 1.27 | 0.03 | 1.56 | C02301 |
| 35 | pos | 566.38 | 6.55 | PC 20:0; PC(10:0/10:0) | 0.40 | 0.01 | 3.54 | C00157 |

**Table 4 Differential metabolites in QPSM vs Aged group**

| **NO.** | **ID** | **MZ** | **RT** | **Metabolite** | **Ratio** | ***P*** | **VIP** | **Kegg ID** |
| --- | --- | --- | --- | --- | --- | --- | --- | --- |
| 1 | neg | 102.06 | 0.73 | 2-Aminoisobutyric acid | 0.70 | 0.03 | 1.95 | C03665 |
| 2 | neg | 103.04 | 1.06 | (R)-3-Hydroxybutyric acid | 2.15 | 0.02 | 2.81 | C01089 |
| 3 | neg | 128.03 | 0.73 | Pyroglutamic acid | 0.77 | 0.02 | 1.67 | C01879 |
| 4 | neg | 128.03 | 1.09 | Pyroglutamic acid | 0.75 | 0.02 | 1.73 | C01879 |
| 5 | neg | 133.01 | 9.19 | D-(+)-Malic acid | 1.96 | 0.04 | 2.46 | C00497 |
| 6 | neg | 146.05 | 0.73 | L-Glutamic acid | 0.67 | 0.02 | 2.15 | C00025 |
| 7 | neg | 168.03 | 0.73 | 2-Methoxy-5-nitrophenol | 0.68 | 0.01 | 2.15 | C17385 |
| 8 | neg | 173.01 | 0.86 | Trifluoroacetic acid | 0.84 | 0.04 | 1.08 | NA |
| 9 | neg | 173.10 | 1.17 | L-(+)-Arginine | 0.80 | 0.02 | 1.44 | C00062 |
| 10 | neg | 184.00 | 1.09 | Phosphoserine | 0.76 | 0.05 | 1.33 | NA |
| 11 | neg | 192.05 | 2.83 | L-Glutamic acid | 0.09 | 0.01 | 4.10 | C00025 |
| 12 | neg | 202.11 | 2.52 | N-lactoyl-Leucine | 1.53 | 0.01 | 1.61 | NA |
| 13 | neg | 239.08 | 0.77 | Allose | 0.79 | 0.03 | 1.59 | C01487 |
| 14 | neg | 243.06 | 2.29 | Uridine | 0.81 | 0.02 | 1.32 | C00299 |
| 15 | neg | 243.20 | 4.38 | 3-Hydroxymyristic acid | 1.87 | 0.01 | 2.48 | NA |
| 16 | neg | 243.06 | 1.62 | Uridine | 0.78 | 0.02 | 1.50 | C00299 |
| 17 | neg | 251.20 | 5.80 | D6-Ambrettolide | 1.59 | 0.03 | 2.07 | NA |
| 18 | neg | 253.22 | 7.17 | Palmitelaidic acid | 1.68 | 0.02 | 2.13 | NA |
| 19 | neg | 279.20 | 4.10 | 2,6-Di-tert-butyl-4-methylphenol | 0.51 | 0.02 | 1.90 | C14693 |
| 20 | neg | 285.21 | 3.64 | Hexadecanedioic acid | 1.56 | 0.00 | 2.20 | C19615 |
| 21 | neg | 317.25 | 7.00 | 5-(8-Pentadecenyl)-1,3-benzenediol | 0.73 | 0.04 | 1.58 | C10770 |
| 22 | neg | 319.23 | 4.82 | 12 Hydroxy arachidonic acid | 0.45 | 0.04 | 2.15 | NA |
| 23 | neg | 355.26 | 7.13 | Tetracosahexaenoic acid | 0.70 | 0.02 | 1.38 | NA |
| 24 | neg | 357.28 | 7.65 | Tetracosapentaenoic acid (24:5n-6) | 0.72 | 0.02 | 1.39 | NA |
| 25 | neg | 424.25 | 4.00 | LysoPE 14:0 | 1.42 | 0.04 | 1.30 | C04438 |
| 26 | neg | 544.27 | 3.74 | LysoPS 20:4; LysoPS 20:4 | 0.79 | 0.01 | 1.24 | C05974 |
| 27 | neg | 555.27 | 3.86 | LysoPG 22:6; LysoPG 22:6 | 0.61 | 0.01 | 2.23 | C05980 |
| 28 | neg | 582.38 | 5.51 | LysoPC 18:0 | 0.77 | 0.01 | 1.29 | C04230 |
| 29 | neg | 804.61 | 4.97 | Plasmenyl-PC 35:0; PC(P-19:0/16:0) | 1.36 | 0.01 | 1.17 | C00958 |
| 30 | neg | 824.58 | 6.61 | Plasmenyl-PC 36:4; PC(P-16:0/20:4) | 0.58 | 0.03 | 2.14 | C00958 |
| 31 | neg | 865.50 | 6.64 | PG 44:12; PG(22:6/22:6) | 0.42 | 0.01 | 2.35 | C00344 |
| 32 | pos | 148.06 | 0.71 | L-Glutamic acid | 0.74 | 0.03 | 1.58 | C00025 |
| 33 | pos | 148.06 | 1.08 | L-Glutamic acid | 0.64 | 0.01 | 2.14 | C00025 |
| 34 | pos | 175.01 | 7.94 | Phenylacetic acid | 1.29 | 0.00 | 1.71 | C07086 |
| 35 | pos | 185.03 | 1.14 | L-Glutamine | 0.79 | 0.03 | 1.30 | C00064 |
| 36 | pos | 186.02 | 0.74 | L-Glutamic acid | 0.71 | 0.04 | 1.87 | C00025 |
| 37 | pos | 203.22 | 2.26 | Spermine | 0.76 | 0.01 | 1.53 | C00750 |
| 38 | pos | 234.18 | 3.65 | 1,10-Oxidocalamenene | 1.13 | 0.00 | 1.03 | NA |
| 39 | pos | 245.08 | 1.62 | Uridine | 0.68 | 0.02 | 1.85 | C00299 |
| 40 | pos | 248.15 | 1.92 | 12-Cytisineacetamide | 3.63 | 0.02 | 3.55 | NA |
| 41 | pos | 248.15 | 1.59 | 12-Cytisineacetamide | 5.35 | 0.00 | 4.39 | NA |
| 42 | pos | 284.18 | 2.80 | Acylcarnitine 8:2 | 0.63 | 0.04 | 2.21 | C02301 |
| 43 | pos | 310.20 | 3.05 | Octanoylcarnitine | 0.63 | 0.03 | 2.00 | C02838 |
| 44 | pos | 312.22 | 3.16 | Acylcarnitine 10:2 | 0.67 | 0.02 | 1.94 | C02301 |
| 45 | pos | 346.05 | 0.84 | L-Glutathione, reduced | 1.17 | 0.03 | 1.19 | C00051 |
| 46 | pos | 448.34 | 4.84 | Acylcarnitine 20:4 | 0.73 | 0.01 | 1.57 | C02301 |
| 47 | pos | 472.34 | 4.76 | Acylcarnitine 22:6 | 0.46 | 0.02 | 2.78 | C02301 |
| 48 | pos | 560.31 | 4.95 | 1-Oleoyl-sn-glycero-3-phosphocholine | 1.26 | 0.00 | 1.18 | C04230 |
| 49 | pos | 644.49 | 6.04 | TG 36:6; TG(12:2/12:2/12:2) | 0.81 | 0.01 | 1.13 | C00422 |
| 50 | pos | 884.54 | 6.62 | BMP 44:12; BMP(22:6/22:6) | 0.59 | 0.00 | 2.27 | NA |
